# CD47 blockade-driven necroptosis complements BCL-2 inhibition-driven apoptosis in lymphoid malignancies

**DOI:** 10.1186/s13045-025-01774-3

**Published:** 2026-01-03

**Authors:** Stephen J. F. Chong, Rebecca Valentin, Jing Wang, Fen Zhu, Prafulla C. Gokhale, Benjamin K. Eschle, Filip Garbicz, Kartini Iskandar, Tomasz Sewastianik, Brienne C. Y. Toh, Johany Penailillo, Marisa O. Peluso, Jeremy Zhang, Liam Hackett, Mary C. Collins, Timothy Z. Lehmberg, Ammar Adam, Li Zhang, Caroline M. Armet, Matthew Rausch, Benjamin H. Lee, Pamela M. Holland, Vito J. Palombella, Alison M. Paterson, Li Ren Kong, Elisa ten Hacken, Jennifer L. Guerriero, Charles Herbaux, Catherine J. Wu, Wee Joo Chng, Shazib Pervaiz, Carsten U. Niemann, Ruben D. Carrasco, Matthew S. Davids

**Affiliations:** 1 Department of Medical Oncology, Dana-Farber Cancer Institute, Boston, USA; 2 Cancer Science Institute of Singapore, Yong Loo Lin School of Medicine, N2CR, NUS, Singapore, Singapore; 3 Department of Physiology, Yong Loo Lin School of Medicine, National University of Singapore (NUS), NUS Centre for Cancer Research (N2CR), NUS, Singapore, Republic of Singapore; 4 Department of Hematology, Rigshospitalet, Copenhagen, Denmark; 5 Experimental Therapeutics Core and Belfer Center for Applied Cancer Science, Dana-Farber Cancer Institute, Boston, MA USA; 6 Department of Oncologic Pathology, Dana-Farber Cancer Institute, Harvard Medical School, Boston, MA USA; 7 Surface Oncology, Inc., Cambridge, MA USA; 8 Cancer Discovery and Regenerative Medicine Programme, Lee Kong Chian School of Medicine, Nanyang Technological University, Cancer Science Institute of Singapore, NUS, Singapore, Singapore; 9 Department of Surgery, Brigham and Women’s Hospital, Boston, MA USA; 10 Institute of Clinical Medicine, University of Copenhagen, Copenhagen, Denmark

**Keywords:** CD47, Necroptosis, Apoptosis, Phagocytosis, BH3 profiling, Venetoclax resistance, Lymphoid malignancy

## Abstract

**Background:**

Immune checkpoint blockade of CD47 has shown promising results in lymphoid malignancies, with its effects attributed to enabling tumor-cell phagocytosis. However, alternate cytotoxic cell death mechanisms have been reported, potentially contributing to the overall anti-tumor activity. Although previous studies have highlighted a mechanism of caspase-independent cell death, this mechanism has yet to be well-characterized, thereby warranting further investigation to comprehensively understand the anti-tumor mechanism of CD47 blockade to facilitate optimal drug partner selection for combination therapy.

**Methods:**

The fully humanized anti-CD47 monoclonal antibodies, SRF231, magrolimab, as well as a mouse monoclonal anti-CD47 antibody, B6H12, were used. Multiple cell death mechanisms were evaluated including apoptosis, autophagy and necroptosis by using customized Hoechst/Annexin V, the precision medicine technique BH3 profiling, as well as standard experimental techniques – flow cytometry, siRNA and CRISPR Cas9 genetic manipulation, Western blotting, and immunohistochemistry. These techniques were used on a comprehensive range of lymphoid malignant models including diffuse large B-cell lymphoma, Burkitt lymphoma, and T-acute lymphoblastic leukemia cell lines, patient primary chronic lymphocytic leukemia cells, as well as lymphoid cell-line derived and patient-derived xenograft mice, to elucidate the mechanism of cell death by CD47 blockade and to identify the optimal drug partners for treatment combination.

**Results:**

We demonstrate that the anti-CD47 antibodies SRF231, magrolimab, and B6H12 eliminated tumor cells from various in vitro and in vivo lymphoid malignant models via the activation of the RIPK1/MLKL/necroptotic pathway. Moreover, the BH3 profiling technique distinguished two different lymphoid malignant models that respond differently to the BCL-2 inhibitor venetoclax when combined with SRF231; one highlighting the effective yet distinct mechanisms of SRF231-induced necroptosis and venetoclax-induced apoptosis in models that were specifically and/or highly dependent on BCL-2 for survival, while the other implicating venetoclax as a counterproductive partner with SRF231 in models that were not dependent on BCL-2 for survival or were not responsive to venetoclax treatment.

**Conclusion:**

Collectively, this study unravels a novel, non-canonical cell death mechanism of targeting CD47 by activating necroptosis, and provides evidence and rationale for further evaluation of a therapeutic strategy of combining CD47 blockade with and without apoptotic inducers for suitable patients with lymphoid malignancies.

**Graphical Abstract:**

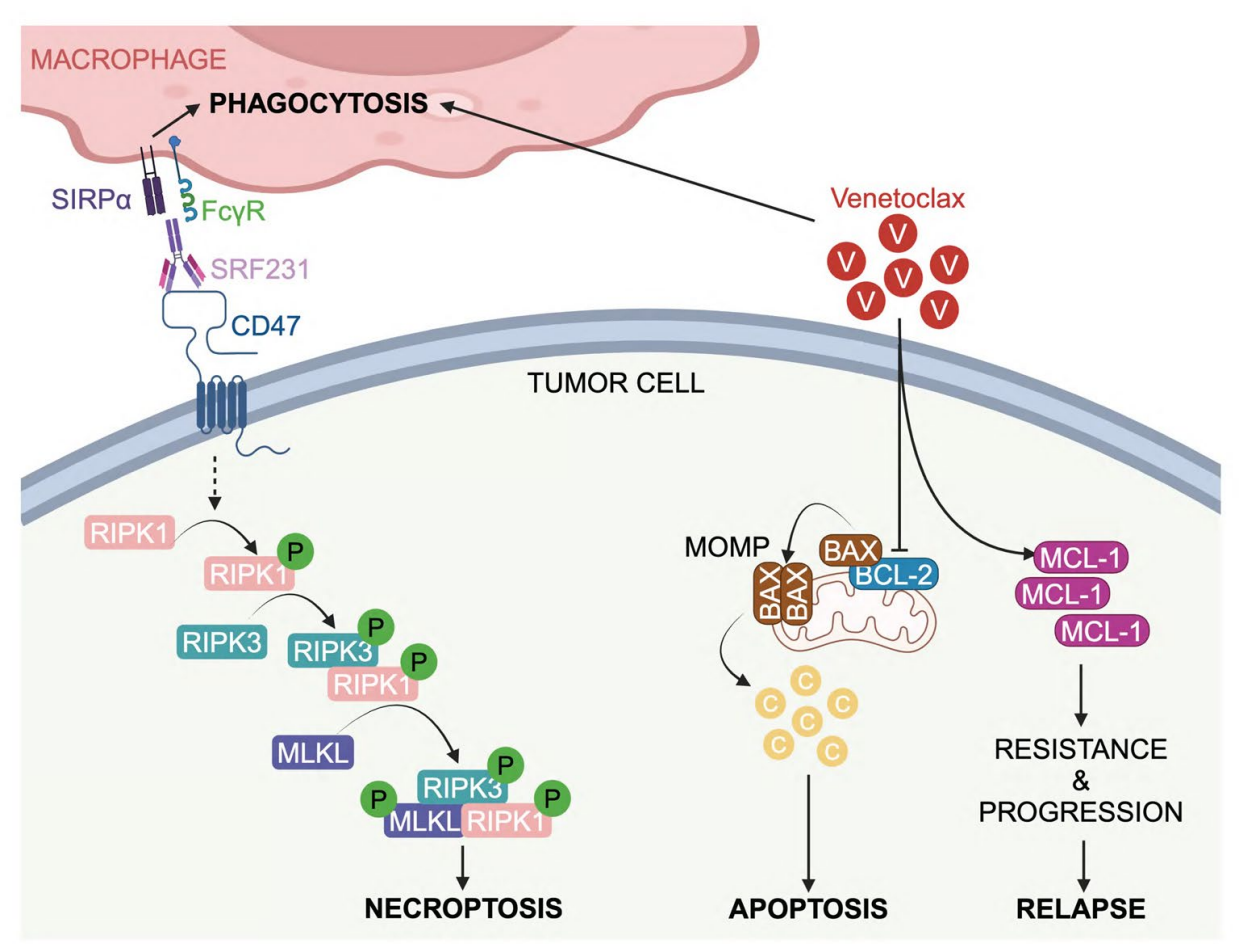

**Supplementary Information:**

The online version contains supplementary material available at 10.1186/s13045-025-01774-3.

## Introduction

Blockade of immune checkpoints has become one of the most promising therapeutic approaches in cancer. While agents targeting T cell immunity such as antibodies against cytotoxic T-lymphocyte-associated protein-4 (CTLA-4), programmed death-1 (PD-1) and its ligand (PD-L1) have been highly effective in certain solid tumors and lymphoma subtypes, their efficacy in other hematologic malignancies has been limited [1–7]. These established immune checkpoint blockade antibodies have focused on augmenting the anti-tumor activity of the adaptive immune system; however, alternative immune checkpoints in the innate immune system have also been identified as promising targets.

One such target is CD47, a macrophage checkpoint protein commonly expressed on tumor cells, that serves as a “don’t-eat-me” signal for macrophage-mediated phagocytosis. CD47 binds to signal regulatory protein alpha (SIRPα) expressed on myeloid cells, thereby counteracting pro-phagocytic signals and facilitating evasion of innate immune surveillance. High levels of CD47 protein expression have been widely reported across different tumor types and are an independent negative prognostic factor in multiple cancers [8–17], providing a strong rationale for disruption of the CD47/SIRPα axis as a therapeutic intervention. SRF231 is a fully human, IgG4 isotype, anti-CD47 monoclonal antibody (mAb) that disrupts the CD47/SIRPα interaction. In pre-clinical studies, SRF231 promotes macrophage-mediated phagocytosis of tumor cells across a broad range of tumor cell lines and primary acute myeloid leukemia (AML) cells without inducing hemagglutination or red blood cell (RBC) phagocytosis [18]. In addition, SRF231 induces caspase-independent cell death in a manner dependent on CD32a, an FcγR predominantly expressed on myeloid cells [18, 19]. SRF231 has been tested in a first-in-human phase 1 clinical trial across multiple tumor types (NCT03512340) [20], yet the precise mechanism of its ability to cause novel phagocytotic-independent tumor cell death remains unknown.

Resistance to cell death via the dysregulation of the mitochondrial apoptotic pathway is a common feature of chemoresistance. This is often associated with changes in the expression, function or survival dependences of the apoptotic regulators, known as the B-cell leukemia/lymphoma-2 (BCL-2) family of proteins [21–30]. BH3 profiling is a functional precision medicine technique that probes the mitochondria with BH3-only peptides to assess the proximity of cells to the threshold of apoptosis (i.e. mitochondrial priming) and the specific pattern of BCL-2 family anti-apoptotic proteins (e.g. BCL-2, MCL-1, BCL-xL, or BFL-1) that the cells depend on for survival [25, 27, 29].

Venetoclax is a first-in-class pro-apoptotic BCL-2 Homology-3 (BH3) mimetic that selectively antagonizes the anti-apoptotic BCL-2 protein, thereby rapidly inducing apoptosis in sensitive tumor cells. Venetoclax was initially found to be highly active in chronic lymphocytic leukemia (CLL) and AML, which led to approvals in those diseases [21, 31, 32]. Although combination approaches of venetoclax with Bruton Tyrosine Kinase (BTK) inhibitors and/or anti-CD20 monoclonal antibodies in CLL or venetoclax with hypomethylating agents in AML have improved outcomes, none of these regimens have proven to be curative, suggesting the persistence of treatment resistant tumor cells [33–36]. Moreover, venetoclax is generally less efficacious in other hematologic malignancies such as non-Hodgkin lymphomas (NHLs) [37, 38]. Therefore, identifying alternative approaches or novel combination partners for venetoclax that complement its ability to induce apoptosis has the potential to improve therapeutic outcomes for a broad population of patients with hematologic malignancies.

Here, we aimed to further delineate the mechanisms underlying anti-CD47-induced cell death by SRF231, and to elucidate through BH3 profiling the suitability and mechanisms underlying the activity of CD47 blockade in combination with venetoclax in lymphoid malignancies.

## Methods

### Primary cells and cell lines

Human peripheral blood mononuclear cells (PBMCs) were collected from patients with CLL and healthy donors, and were isolated from whole blood by density gradient centrifugation over Ficoll-Paque (GE Healthcare), viably frozen in FBS (Gibco, Thermo Fisher Scientific) supplemented with 10% DMSO until the time of analysis. Upon thawing, cells were cultured in RPMI 1640 medium supplemented with 10% FBS, 100 U/ml penicillin, and 100 µg/ml streptomycin (Gibco) (R10). Ri-1 (Sigma-Aldrich, cat# 96090512), Raji (ATCC, cat# CCL-86), Jurkat (Clone E6-1, ATCC, cat# TIB-152) and Jurkat CD47 CRISPR/Cas9-knockout (KO) (Applied StemCell), OCI-Ly3 (DSMZ, cat# ACC761), OCI-Ly1, OCI-Ly1-R, TMD8 (Wu Lab, DFCI), MOLM14-S (Chng Lab, CSI, NUS), MOLM14-R, HL60 (Pervaiz Lab, NUS) cell lines were maintained in R10. OCI-Ly1-R and MOLM14-R were previously generated from OCI-Ly1-S and MOLM14-S, respectively [25, 39]. All cell lines were tested negative for mycoplasma and authenticated.

### Reagents and antibodies

Reagents were procured as follows: SRF231 (Surface Oncology), hIgG4 Isotype control (Surface Oncology), venetoclax (MedChemexpress Co., #HY-15531). RosetteSep Human Monocyte Enrichment Cocktail (Stem Cell Technologies, #15068), APC mouse anti-human CD14 (BD Biosciences, #555399), APC mouse anti-human CD206 (BD Biosciences, #550889), Magrolimab (ProSci, #10–950), mouse IgG (BD Biosciences, #554721), B6H12 (BD Biosciences, #556043), Recombinant human M-CSF (Life Technologies, #PHC9501), Cell trace CFSE (Life Technologies, #C34554), Zombie Violet fixable viability kit (Biolegend, #423113), Z-VAD-FMK (Santa Cruz Biotechnology, #sc-3067), BAPTA/AM (Santa Cruz Biotechnology, sc-202488), GSK’872 (Selleckchem, #S8465), Necrostatin-1 (Selleckchem, #S8037), Mounting medium with DAPI (ThermoFisher, #P36931), Alexa Fluor-488 anti-Rabbit IgG (Invitrogen, #A11034), Hoechst (Invitrogen, #H3570), Alexa Fluor 488-Annexin V (Life Technologies, #A13201).

The primary antibodies used for Western blot analyses were as follows: RIPK1-phospho-Ser166 rabbit antibody (Cell Signaling Technology, #65746), RIPK1 rabbit antibody (Cell Signaling Technology, #3493), MLKL-phospho-ser358 rabbit antibody (Cell Signaling Technology, #91689), anti-MLKL rabbit antibody (Cell Signaling Technology, #14993), β-Actin mouse antibody (Santa Cruz Biotechnology, #sc-47778), caspase 3 rabbit antibody (Cell Signaling Technology, #9662), anti-LC3 rabbit antibody (Cell Signaling Technology, #4108), anti-ERK rabbit antibody (Cell Signaling Technology, #4695), anti-PGAM rabbit antibody (Abcam, #ab126534), anti-rabbit IgG, HRP-linked antibody (Cell Signaling Technology, #7074), anti-mouse IgG, HRP-linked antibody (Cell Signaling Technology, #7076). The primary antibodies used for immunohistochemistry were as follows: MLKL-phospho-Ser358 rabbit antibody (Abcam, #ab187091), MLKL-phospho-Thr357 mouse antibody (Novus Biologicals, #MAB91871) and RIPK1-phospho-ser166 rabbit antibody (Thermofisher, #PA5-104645). The primary antibodies used for immunocytochemistry were as follows: RIPK1-phospho-ser166 rabbit antibody (Thermofisher, #PA5-104645) and MLKL-phospho-ser358 rabbit antibody (Cell Signaling Technology, #91689).

### Tumor xenograft studies

#### Cell line-derived xenograft model

Animal studies were conducted at Surface Oncology and Dana-Farber Cancer Institute according to guidelines established by the internal and external Institutional Animal Care and Use Committee. Cancer cells (Ri-1: 1 × 10^7^, HL60: 5 × 10^6^) in a 1:1 mixture of RPMI and Matrigel (BD Biosciences) were subcutaneously implanted into the right flank of female CB17-SCID mice (Charles River Laboratories, Massachusetts, USA). Antibody treatments were initiated when tumors averaged 200–300 mm^3^ (Ri-1) or 100–200 mm^3^ (HL60) following randomization, for one cycle. Animals were monitored, and body weight and tumor volume were measured twice weekly by electronic callipers (length x width^2^) x 0.52. Complete response (CR) was determined when subcutaneous tumor volume was not detected through electronic calliper or palpation.

### Patient-derived xenograft (PDX) model

The in vivo studies with a patient-derived xenograft model were performed in a diffused large B-cell lymphoma (DLBCL) PDX model (DFBL-18689) from the Public Repository of Xenografts (PRoXe) [40]. Female Nod.Cg-*Prkdc*^*scid*^*IL2rg*^*tm1Wjl*^/SzJ (NSG) mice [41, 42] (Strain #005557, The Jackson Laboratory) were implanted with approximately 1 million cells intravenously before reaching 8-weeks of age. The tumor burden in mouse peripheral blood was monitored by flow cytometry for human CD45+ cells. For drug treatments, Isotype control or SRF231 were diluted in PBS and administered intraperitoneally (IP) and venetoclax was formulated with 10% ethanol, 60% Phosal 50PG and 30% PEG400 and administered orally.

### Scaffolding/immobilization of antibody and protein G-based cytotoxicity assay

Scaffolding or immobilization of antibodies was performed using Protein G-coated plates (Thermo Fisher, #15156 or #15131). These plates were designed to orient antibodies for maximum antigen-binding capability, retain antibody activities, ensure minimal variation, and reduce non-specific binding. Plates were first washed three times with PBS and coated overnight with antibody diluted in PBS at their specified concentrations. Following additional washing with PBS, plates were incubated with the target cells and/or drug (venetoclax) at 37 °C, 5% CO_2_ at specific timepoints. After Annexin V (AnnV)/Hoechst staining, cells were fixed with 4% paraformaldehyde, neutralized with N2 buffer (1.7 M Tris, 1.25 M glycine pH 9.1) and analyzed using a BD Fortessa with 96-well HTS plate-reader. Flow cytometry gating for AnnV/Hoechst assay is shown in supplemental Fig. 1A. Note that cells stain AnnV-positive when undergoing necroptosis [43–46]. CellTiter-Glo^®^ Luminescent Cell Viability Assay (Promega, #G7570) or Caspase-Glo^®^ 9 Assay (Promega, #G8211) was used following the manufacturer’s instructions and analyzed on a Tecan plate reader.

### Transfection via electroporation

The Neon™ electroporation transfection system and kit (#MPK10096) were used for the transfection of ON-TARGETplus siRNA against human MLKL (#J-005326-05-0020) at a concentration of 50nM and cell density of 1 × 10^7^. Cells were first washed with PBS and re-suspended in Resuspension buffer R. SiRNA was then added to cell suspension before electroporation using the 100 µl Neon transfection tip and pipette at a setting of 1150 V/30ms for 2 pulses. Cells were then seeded in 10 ml antibiotic-free RPMI supplemented with 10% FBS and 2mM L-glutamine for 48 h at 37 °C, 5% CO_2_ prior to subsequent experiments.

### Immunoblotting

Immunoblotting was performed as previously described [29].

### Macrophage generation and phagocytosis assay

**Fig. 1:**
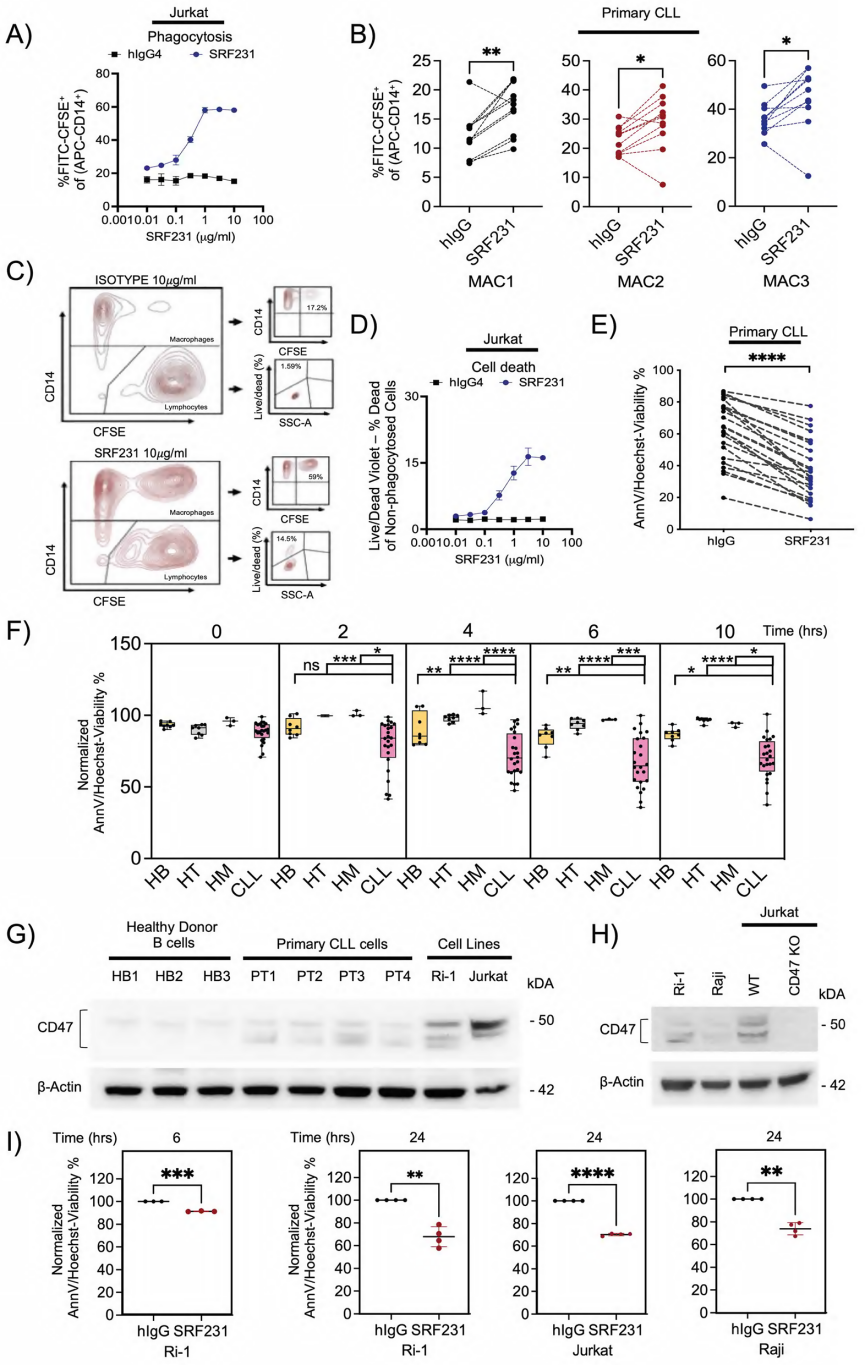
Targeting CD47 with SRF231 induces phagocytosis and cell death in lymphoid malignant cells. **A.** Phagocytosis induction evaluated in the presence of 2-hour treatment with SRF231 or hIgG4 isotype in Jurkat cells cocultured with hMDM (*n* = 2). **B.** Phagocytosis induction evaluated in the presence of 2-hour treatment with SRF231 or hIgG4 isotype in 11 CLL patient samples cocultured with hMDMs from 3 different donors (MAC1—MAC3). Reported *P* values were calculated by paired Student’s *t* test. **C.** Diagram showing gating strategy – Phagocytosis is identified as the CD14^+^ population with uptake of CFSE^+^ lymphocytes. SRF231-mediated cell death is identified as the non-phagocytosed CD14^−^ cells with CFSE and Violet Live/Dead uptake. Violet Live/Dead (Zombie Violet™) is an amine-reactive fluorescent dye that stains cells that have compromised membranes as compared to AnnV stain that binds phosphatidylserine. Representative flow cytometry analyses showing the effect of hIgG4 isotype (10 µg/ml, top panel) and SRF231 (10 µg/ml, bottom panel) on phagocytosis and cell death of non-phagocytosed cells, cocultured with hMDMs. **D.** Population of non-phagocytosed Jurkat cells following treatment with either hIgG4 isotype or SRF231 were analyzed by flow cytometry. Tumor cell death denoted by % of death of non-phagocytosed cell population (*n* = 2). **E**. Cells from 24 CLL patient samples were subjected to Protein G-bound SRF231 (10 µg/ml) and cell viability were measured by AnnV/Hoechst cell death assay. Decrease in percent live (AnnV-/Hoechst+) cells in SRF231 vs. hIgG4 isotype treatment was reported. Reported *P* values were calculated by paired Student’s *t* test. **F.** Healthy B cells (HB, *n* = 8), healthy T cells (HT, *n* = 8), healthy monocytes (HM, *n* = 3) and PBMC cells from CLL patients (CLL, *n* = 24) were subjected to Protein G-bound SRF231 or hIgG4 isotype treatment (10 µg/ml). Cell death induction was measured using AnnV/Hoechst assay at 0, 2, 4, 6 and 10 h, and data presented were % live cells of SRF231 normalized to their respective hIgG4 isotype. Reported *P* values were calculated by Sidak’s multiple comparison test. **G-H.** Western Blot showing CD47 and β-actin protein expressions in primary HB and CLL cells, Ri-1, Raji and Jurkat (Wild type and CD47 Knockout) cells. **I.** Cell death inductions of Ri-1 (*n* = 3, 6 h; *n* = 4, 24 h), Jurkat (*n* = 4) and Raji (*n* = 4) cells were measured following Protein G-bound SRF231 incubation with AnnV/Hoechst assay at indicated timepoints. Reported *P* values were calculated by paired Student’s *t* test

CD14^+^ monocytes were isolated from whole blood from healthy donors via negative selection using RosetteSep Human Monocyte Enrichment Cocktail (Stem Cell Technologies) and density gradient centrifugation over Ficoll-Paque (GE Healthcare). Isolated monocytes were then viably frozen in FBS supplemented with 10% DMSO until the time of analysis. Primary monocytes were then thawed and cultured for 6 days in the presence of 100 ng/ml hM-CSF (Life Technologies). Primary cells from CLL patients or cell lines were labeled with CellTrace CFSE (Life Technologies) and incubated with macrophages in a target: effector-ratio of 2:1, and treated with SRF231 or hIgG4 Isotype (10 µg/ml) for 2 h at 37 °C. Cells were washed and re-suspended in CD14-APC or CD206-APC (BD Biosciences), diluted in FACS buffer (500 ml PBS + 10 ml FBS + 2 ml 0.5 M EDTA, Life Technologies/Fisher Scientific) and analyzed on a BD Fortessa using a 96-well HTS plate-reader. For combined phagocytosis and cell death assessment, Zombie Violet™ Fixable Viability Kit (Biolegend) was added together with CD14-APC. The Zombie Violet™ Fixable Viability Kit is an amine-reactive fluorescent dye that is non-permeant to live cells but permeant to cells that have compromised membranes. Phagocytic index was defined as the percentage of the total population of CD14^+^ or CD206^+^ cells with uptake of CFSE^+^ lymphocytes. Flow cytometry gating for these assays are shown in Fig. 1C and supplemental Fig. S1B.

### BH3 profiling

BH3 profiling was performed as previously described [29]. Cells were suspended in MEB2P buffer (150 mM mannitol, 10 mM HEPES-KOH pH 7.5, 150 mM KCI, 1 mM EGTA, 1 mM EDTA, 0.1% BSA, 5 mM succinate, 0.25% poloxamer 188) prior to analysis. Suspended cells were added to 384-well plates and incubated for 60 min with BH3-only peptides (BIM, BAD, MS1, HRK and FS1) or BH3 mimetic drug (ABT-199/venetoclax) combined with 0.002% digitonin for cell membrane permeabilization. Following fixation with 4% paraformaldehyde and neutralization with N2 buffer (1.7 M Tris, 1.25 M glycine pH 9.1), cells were stained overnight with a cocktail of anti–cytochrome *c*–Alexa Fluor 488 (Biolegend, Cat# 612308), anti–CD19-PE/Cy7 (Biolegend, Cat# 302216), anti–CD5-PE (Biolegend, Cat# 300608), and Hoechst 33342 (Invitrogen, Cat# H3570) and analyzed using a BD FACS Fortessa. Flow cytometry data were analyzed using FACS Diva version 8.0.1 (BD Pharmingen). Individual analyses were generally performed in triplicate for all conditions. Cytochrome *c* release was used to assess the degree of mitochondrial outer membrane permeabilization in response to each BH3 peptide, which was normalized, relative to cytochrome *c* release with DMSO (0% loss, negative control) and the ion-channel forming peptide alamethicin (100% loss, positive control). BAD peptide was used as measure for BCL-2 and BCL-xL survival dependences, MS1 peptide for MCL-1 survival dependence, HRK peptide for BCL-xL survival dependence, FS1 peptide for BFL1 survival dependence, and venetoclax for BCL-2 survival dependence. The BIM peptide was used to measure overall mitochondrial priming for apoptosis. Increase in cytochrome *c* release in response to BIM BH3 peptide indicates increased mitochondrial priming for apoptosis, while increase in cytochrome *c* release in response to BAD, MS1, HRK, FS1 or venetoclax indicates increased survival dependence on specific anti-apoptotic proteins. Flow cytometry gating is shown in supplemental Fig. S1C.

### Immunocytochemistry

Protein G-bound isotype or SRF231-treated cells were collected and Cyto-spun onto the slide (500,000 cells/slide) using Cytospin 3 Shandon. Cells were then fixed using 4% paraformaldehyde and permeabilized with 0.2% TritonX-100/PBS. Following 2 washes with PBS, cells were blocked with 5% FBS in PBS with 1% BSA. Primary antibodies (p-RIPK1 or p-MLKL, see reagents and antibodies section) were used in 1:100 dilution and incubated for 2 h at RT and then incubated with a Alexa Fluor-488 anti-Rabbit IgG for 1 h. Cells were then washed with PBS and mounted with Mounting Medium containing DAPI stain. Cells were subsequently analyzed under a confocal fluorescence microscope (Nikon inverted TE2000 microscope, 60x Plan-fluor DIC NA 0.85 w/collar objective).

### Immunohistochemistry (IHC)

Tumor tissues harvested from in vivo mouse work were fixed in 10% formalin, embedded in paraffin, cut into 4 μm sections, deparaffinized and dehydrated. The sections underwent heat-mediated antigen retrieval and blocking. They were then incubated with primary antibodies (p-MLKL S358, ab187091, 1:50 or p-MLKL T357, MAB91871, 1:250 or p-RIPK1 S166, PA5-104645, 1:100), washed and incubated with secondary anti-rabbit HRP-conjugated antibody, developed with chromogen, and mounted. Images were acquired using a Leica DM2000 microscope (Leica Microsystems). For image quantification, slides were scanned using Vectra 2 Intelligent Slide Analysis System (Caliper LifeSciences). Intensity of p-MLKL T357, S358, and p-RIPK1 S166 staining was assessed in 6 representative region of interests (ROIs) per sample (*n* = 3 per treatment) using HALO Image Analysis Software and Multiplex IHC module (Indica Labs).

### Figures and statistical analysis

Data were presented as mean ± SD, unless otherwise specified in the figure legend. Data plotting and statistical analyses were performed using GraphPad Prism software. When comparing two groups, two-tailed unpaired/paired Student’s *t* test or Welch *t* test was used, and *p* < 0.05 was considered significant. Multiple groups were compared using one-way or two-way ANOVA with either Sidak’s or Dunnett’s correction for repeated measures, where *p < 0.05; **p < 0.01; ***p < 0.001; ****p < 0.0001; ns, not significant. For survival data, the Kaplan-Meier method was used to estimate survival functions and log-rank test (Mantel-Cox) was applied to group comparisons.

## Results

### Targeting CD47 with SRF231 induces phagocytosis and cell death in lymphoid malignant cells

To evaluate the canonical role of CD47 blockade in inducing phagocytosis by SRF231, we first developed a phagocytosis assay using human monocyte-derived macrophages (hMDMs) generated by M-CSF treatment of monocytes from the blood of healthy donors. hMDMs were cocultured with CFSE-labeled target cells and exposed to CD47 mAb SRF231, or hIgG4 isotype control. To track the degree of phagocytosis, macrophages were stained with fluorescently-labeled anti-CD14, and the percentage of CD14:CFSE double-positive cells (representing phagocytosed cells) was determined (Suppl. Figure S2A). SRF231 treatment (2-hour) led to a drastic increase in phagocytosis of human Jurkat leukemia cells by hMDM (Fig. 1A). Increased phagocytosis was also observed with a more conventional fluorescently-labeled anti-CD206 macrophage surface marker in Jurkat and Ri-1 lymphoma cells (Suppl. Figure S2B). Similarly, increased phagocytosis of primary cells derived from the peripheral blood of CLL patients (*n* = 11) by 3 different donor hMDMs was observed with SRF231 treatment (2-hour) (Fig. 1B). Interestingly, as CD47 antagonists have been reported to induce cell death in a manner associated with a unique caspase-independent cell death pathway in certain hematologic malignancies [18, 47, 48], we proceeded to investigate whether cell death is also observed in Jurkat cells that were non-phagocytosed via the specific gating shown in Fig. 1C. Indeed, we observed a dose-dependent increase in Jurkat cell death in the non-phagocytosed population (Fig. 1D). As our previous work demonstrates that the Fc receptor of the macrophage acts as a scaffold for the Fc region of the SRF231 antibody to drive CD47-mediated cell death [18], we further ascertained this non-canonical role of SRF231 in tumor cell death induction by mimicking the antibody scaffolding by hMDM via SRF231 immobilization on protein G-coated plates prior to the addition of primary CLL cells (in the absence of hMDM co-culture). Primary CLL cells incubated with SRF231 for 10 h showed a clear and significant decrease in cell survival as compared to hIgG4 isotype control (Fig. 1E). Collectively, these findings suggest a non-canonical cytotoxicity that is independent of phagocytosis.

High expression of CD47 has previously been reported in CLL, and is associated with shorter event-free survival [11]. With that in mind, we further tested whether primary CLL cells would be more susceptible to SRF231 treatment as compared to human peripheral blood mononuclear cells (PBMCs) of healthy donors. Using the protein G-coated plates, we observed in a time-course experiment that SRF231 primarily affected the viability of primary CLL cells as early as 6 h, without significantly impacting the viability of normal healthy B and T cells or monocytes (Fig. 1F), hence suggesting a selective effect on malignant lymphoid cells. The higher cell death level observed in primary CLL cells as compared to that of healthy B cells was consistent with the higher expression levels of CD47 and its isoforms observed (Fig. 1G), which were previously shown to possess similar function [49].

We proceeded to re-capitulate the cytotoxic effect of SRF231 in our lymphoid malignant cell lines, which express variable degrees of CD47, in comparison to CRISPR/Cas9-KO CD47 Jurkat cells (Fig. 1G and H). Indeed, the cell death effect of SRF231 was observed more prominently at 24 h in various lymphoid malignant lines of Ri-1, OCI-Ly1 (DLBCL), Raji (Burkitt’s lymphoma) and Jurkat (T-acute lymphocytic leukemia) cells as well as myeloid malignant lines of MOLM14 and HL60 (AML, Fig. 1I, Suppl. Figure S3A). No cell death was previously reported in the CD47 KO Jurkat cells [18]. Of note, these initial experiments informed the timepoints used for subsequent experiments (cell lines – 24 h, primary samples – 6 h).

### SRF231-induced cell death is independent of apoptosis or autophagy

To elucidate the distinct mechanism of SRF231-mediated cell death in malignant lymphoid cells in the absence of hMDM coculture, we tested if SRF231-induced cell death were driven by apoptosis. We inhibited apoptosis by pre-treating primary CLL cells with a pan-caspase inhibitor (Z-VAD-FMK) for 30 min before culturing with scaffolded SRF231. Venetoclax was used as a positive control for apoptosis. The addition of Z-VAD-FMK did not prevent SRF231-induced cell death, whereas the caspase-dependent cell death by venetoclax was significantly abrogated (Suppl. Figure S4A). In addition, we observed that treatment with scaffolded SRF231 in primary CLL cells or Ri-1 lymphoma cells did not induce caspase 3 cleavage as compared to the positive control venetoclax (Suppl. Figure S4B), despite eliciting comparable reduction in cell viability. These data suggest that apoptosis is not a primary mechanism underlying SRF231-induced cell death.

Subsequently, we investigated whether autophagy is involved as a cell death mechanism by pre-treating primary CLL cells with BAPTA, an intracellular Ca^2+^ chelator and autophagy inhibitor [50], for 30 min before culturing with scaffolded SRF231. Treatment with BAPTA did not prevent SRF231-induced cell death, suggesting the absence of autophagy-mediated cell death (Suppl. Figure S5A). This is also supported by the absence of altered levels of autophagic markers, LC3A/B-I and LC3A/B-II, following SRF231 treatment in primary CLL cells or Ri-1 lymphoma cells (Suppl. Figure S5B).

### SRF231 induces cell death through activation of the necroptotic pathway in malignant lymphoid cells

Given that apoptosis and autophagy were not the primary pathways of SRF231-induced cell death, we next sought to investigate if the underlying mechanism of SRF231-mediated cell death could be necroptosis, a regulated form of necrosis. As necroptosis involves the reduction of cellular ATP level [51–54], we first measured the ATP levels of Ri-1 and primary CLL cells following scaffolded SRF231 treatment with Celltiter-Glo luminescence, an assay that determines the number of viable cells based on the quantification of ATP levels. We observed that SRF231 treatment reduced ATP levels in Ri-1 and primary CLL cells (Suppl. Figure S6A).

**Fig. 2:**
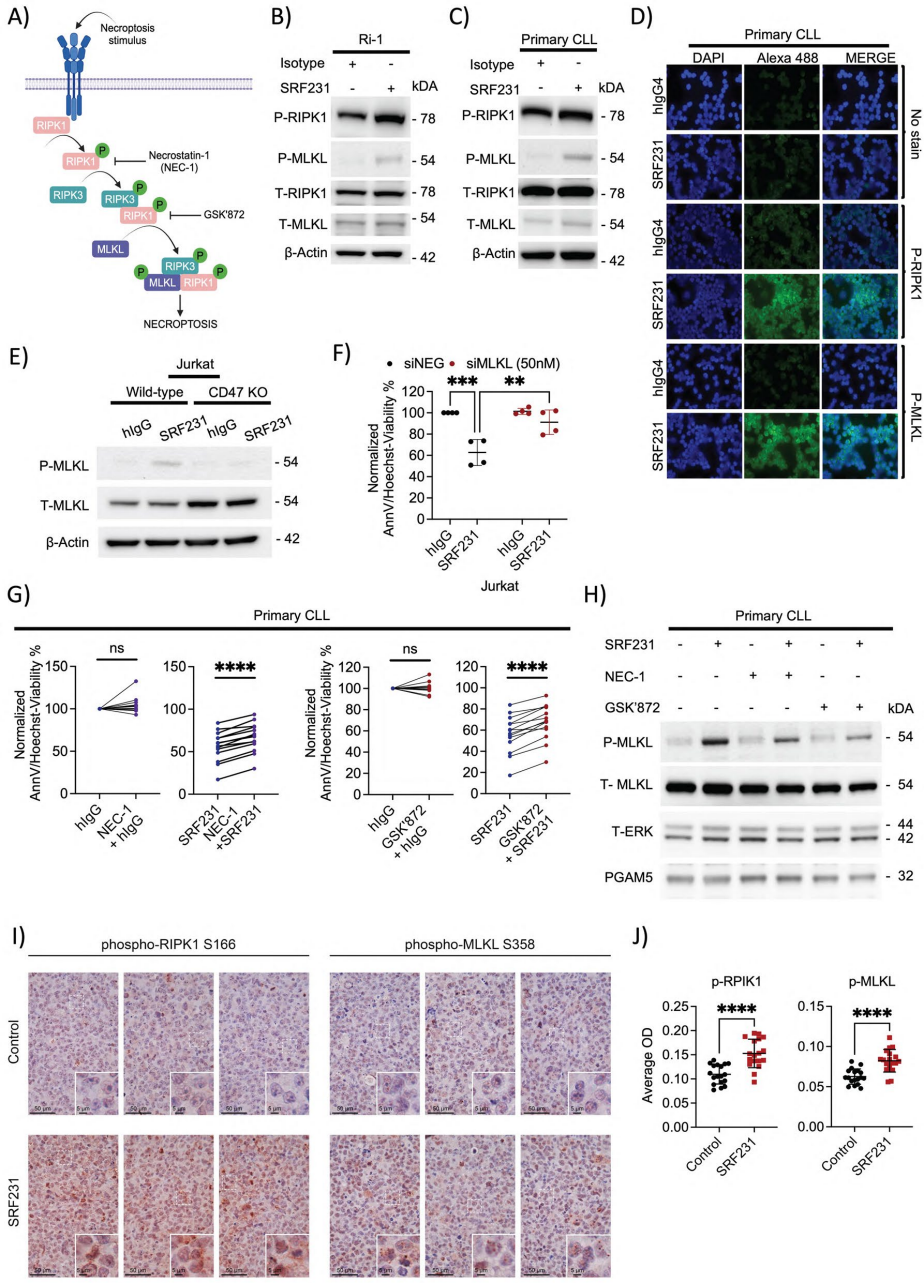
SRF231 induces cell death through necroptotic pathway. **A**. Schematic illustration of necroptosis signalling activation involving the phosphorylation of key proteins such as RIPK1/RIPK3 and MLKL as well as their respective inhibitors. Created in BioRender. Chamberlain, S. (2026) https://BioRender.com/toqddow. **B-C.** Western Blot showing the increased phosphorylation of RIPK1 (S166) and MLKL (S358) after Protein G-bound SRF231 treatment of Ri-1 cells (10 µg/ml, 24 h) or primary CLL cells (10 µg/ml, 6 h). **D**. Images of 6-hour hIgG4 isotype and SRF231 treated primary CLL cells displaying the intensity of p-RIPK1 and p-MLKL (all green), DAPI (blue) and merge using confocal microscopy (60x). **E.** Western blot showing the p-MLKL of wild-type Jurkat and CD47-KO Jurkat cells following treatment with Protein G-bound hIgG or SRF231 (10 µg/ml, 24 h). **F.** Cell viability of Jurkat cells treated with Protein G-bound hIgG or SRF231 (10 µg/ml, 24 h) following 48-hour siMLKL was measured with AnnV/Hoechst assay (*n* = 4). Reported *P* values were calculated by Sidak’s multiple comparison test. **G.** Effect of 1-hour pre-treatment with RIPK3 inhibitor (GSK’872, 50 nM) or RIPK1 inhibitor (Necrostatin-1, 1 µM) on 6-hour Protein G-bound SRF231-induced cell death in 14 patient samples, was measured by AnnV/Hoechst assay. Reported *P* values were calculated by paired two-tailed Student’s *t* test. **H.** Western blot showing the effect of 1-hour pre-treatment with RIPK3 inhibitor (GSK’872, 50 nM) or RIPK1 inhibitor (NEC-1, 1 µM) followed by 6-hour treatment with Protein G-bound SRF231 (10 µg/ml) on p-MLKL in primary CLL cells. Total ERK and PGAM5 were used as loading controls. **I.** p-RIP1K and p-MLKL immunohistochemistry staining of hIgG control- or SRF231-treated formalin-fixed paraffin embedded tissues from subcutaneously implanted Raji tumors in CB17.SCID mice. **J.** Quantification of 6 representative region of interests (ROI) per sample of p-RIP1K and p-MLKL IHC staining in Fig. 2I. (*n* = 3 per treatment). Reported *P* values were calculated by unpaired two-tailed Welch’s *t* test. OD: Optical Density

In addition, the signaling pathway of necroptosis involves the phosphorylation-driven activation of receptor-interacting protein kinases 1/3 (RIPK1/RIPK3) and downstream substrate mixed-lineage kinase domain–like pseudokinase (MLKL). The RIPK1/RIPK3/MLKL-containing necroptosome subsequently executes necroptosis (Fig. 2A; [55–60]). To investigate whether SRF231-induced cell death is necroptosis-driven, the levels of phospho-RIPK1 (p-RIPK1) and downstream phospho-MLKL (p-MLKL) were evaluated in Ri-1 and primary CLL cells. Increases in p-RIPK1 and p-MLKL levels were observed in these malignant lymphoid cells after treatment with SRF231 (Fig. 2B and C). We further confirmed these observations using confocal microscopy, where strong increases in p-RIP1K and p-MLKL were observed in primary CLL cells following SRF231 treatment (Fig. 2D).

To confirm that SRF231 induces necroptotic cell death via CD47/MLKL signaling, we demonstrated that CD47-KO Jurkat cells do not display an increased phospho-MLKL following treatment with SRF231, whereas wild-type (WT) Jurkat cells do (Fig. 2E). This is also in line with our previous finding that CD47-KO Jurkat cells failed to undergo cell death as compared to WT Jurkat cells following SRF231 treatment [18]. To further confirm that MLKL is downstream of CD47 blockade-induced cell death, we subsequently knocked down MLKL using siRNA in Jurkat cells (Suppl. Figure S6B) and observed a close-to-complete rescue of SRF231-induced cell death (Fig. 2F). Moreover, we proceeded to re-capitulate these in vitro cell line findings by pre-treating primary CLL cells with inhibitors of key components of the necroptotic pathway: the RIPK1 inhibitor necrostatin-1 (NEC-1) and the RIPK3 inhibitor GSK’872 [61–63] (Fig. 2A), prior to incubation with scaffolded SRF231. Indeed, GSK’872 and NEC-1 consistently inhibited SRF231-induced cell death and p-MLKL in primary CLL samples (Fig. 2G and H).

Importantly, we further demonstrated that necroptosis induction was more prominent by SRF231, as other CD47 antibodies such as magrolimab or B6H12 (which causes RBC agglutination [64, 65] or RBC phagocytosis [18], respectively) had slightly more modest effects on MLKL phosphorylation (Suppl. Figure S7A). Nonetheless, magrolimab-induced MLKL phosphorylation at similar concentration as SRF231 (10 µg/ml) could still induce cell death, while B6H12-induced MLKL phosphorylation and cell death were only comparable at a higher concentration (100 µg/ml) (Suppl. Figure S7 A-S7B). These data therefore indicate that CD47 antibodies in general (i.e. magrolimab and B6H12) could similarly induce necroptosis via MLKL phosphorylation, albeit may require different concentrations, potentially due to factors corresponding to their structural designs or epitope recognitions. Finally, we observed a significant increase in p-RIPK1 and p-MLKL staining via immunohistochemistry (IHC) in tumor samples from xenograft mice treated in vivo with SRF231 (Fig. 2I and J, staining controls displayed in Suppl. Figure S8 A). These data collectively suggest that a major mechanism of CD47 blockade-induced cell death is through CD47/MLKL-mediated necroptosis.

### Through stimulating necroptosis, SRF231 potentiates venetoclax-induced apoptotic cell death in BCL-2 dependent lymphoid malignant cells

**Fig. 3:**
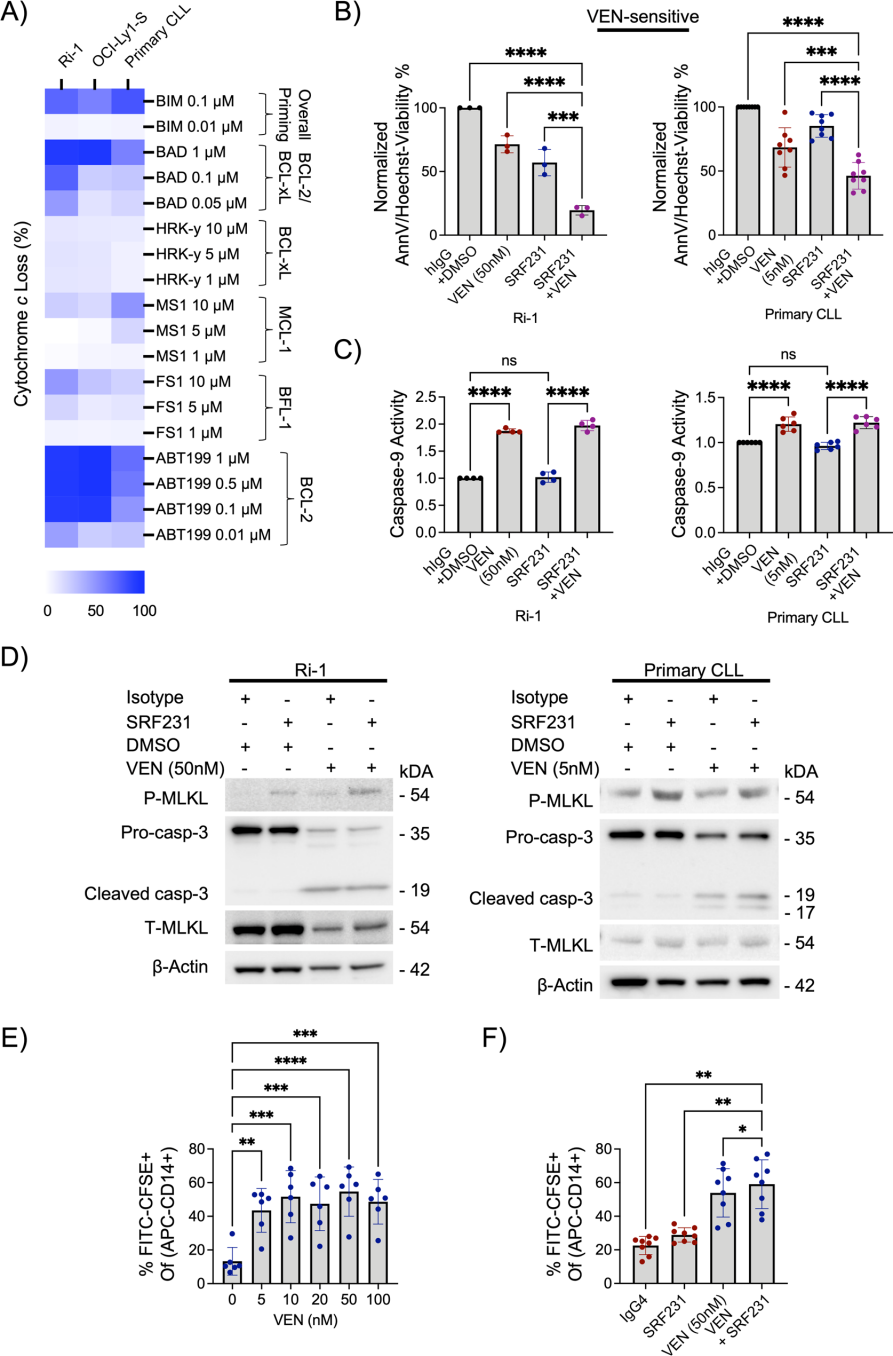
SRF231 and venetoclax (VEN) activate necroptosis, apoptosis and phagocytosis to potentiate cell death in BCL-2 dependent lymphoid malignancies. **A.** BH3 profiling displays anti-apoptotic dependences of lymphoid malignant Ri-1, OCI-Ly1-S (venetoclax sensitive line) and primary CLL cells (average of 3 CLL patients). Right side labels indicating specific anti-apoptotic protein dependences. OCI-Ly1-S is a positive control cell line for high BCL-2 dependence. **B.** Cell viability was measured via AnnV/Hoechst assay for Ri-1 (24 h) and primary CLL (6 h) cells following concurrent treatment with protein G-bound SRF231 and venetoclax. (Ri-1, *n* = 3; Primary CLL, *n* = 8). Reported *P* values were calculated by Sidak’s multiple comparison test. **C.** Fold change in caspase-9 activity measured with Caspase-Glo 9 assay for Ri-1 and primary CLL cells following concurrent treatment with protein G-bound SRF231 and venetoclax. (Ri-1, *n* = 4; Primary CLL, *n* = 6). Reported *P* values were calculated by Sidak’s multiple comparison test. **D**. Western blots showing the increased p-MLKL and caspase 3 cleavage following treatment with Protein G-bound SRF231 or venetoclax or both in Ri-1 cells (SRF231:10 µg/ml, Venetoclax: 50nM, 24 h) or primary CLL cells (SRF231:10 µg/ml, Venetoclax:5nM, 6 h). **(E)** Primary CLL cells from coculture with hMDMs exposed to multiple doses of venetoclax (nM). After 2 h incubation, the degree of phagocytosis was measured (*n* = 6). *P* values were calculated by Dunnett’s multiple comparisons test. **(F)** Induction of phagocytosis of primary CLL cells in coculture with hMDMs exposed to 50 nM venetoclax and 10 µg/ml hIgG4 isotype or SRF231 for 2 h (*n* = 8). *P* values were calculated by Sidak’s multiple comparison test

Our data suggest that SRF231 specifically activates necroptosis. We therefore hypothesized that simultaneously targeting a complementary cell death pathway such as apoptosis, may potentiate cell death in malignant lymphoid cells. We first evaluated the anti-apoptotic protein dependence of these malignant lymphoid cells by BH3 profiling, which confirmed that both Ri-1 and primary CLL cells were highly dependent on BCL-2 for survival, thereby suggesting that they would likely be sensitive to venetoclax, a drug approved for treatment of CLL (Fig. 3A). Indeed, the combination of SRF231 and low doses of venetoclax resulted in significant increase in Ri-1 and primary CLL cell death compared to those of single agent treatments (Fig. 3B).

Venetoclax and SRF231 co-treatment activated two distinct cell death pathways in both Ri-1 and primary CLL cells, including increased caspase 9 activity and cleaved caspase 3 (apoptosis) by venetoclax and p-MLKL (necroptosis) by SRF231 (Fig. 3C and D). We further demonstrate that 2-hour venetoclax treatment enhances phagocytosis in primary CLL cells, even at concentrations as low as 5 nM (Fig. 3E), potentially due to the canonical phosphatidyl-serine upregulation [66].

We next investigated whether the treatment combination of SRF231 and venetoclax could further enhance phagocytosis in these cells. Indeed, we observed an increase in phagocytosis following 2-hour treatment combination of SRF231 and venetoclax in primary CLL cells (Fig. 3F). Although modest, the phagocytotic effect occurred in tandem with SRF231-induced necroptosis and venetoclax-induced apoptosis. Therefore, this treatment combination suggests a triple anti-tumor effect involving the simultaneous activation of three different killing modalities: necroptosis, apoptosis and phagocytosis.

### SRF231 plus venetoclax is highly active in vivo in BCL-2-dependent DLBCL and AML mouse models

**Fig. 4:**
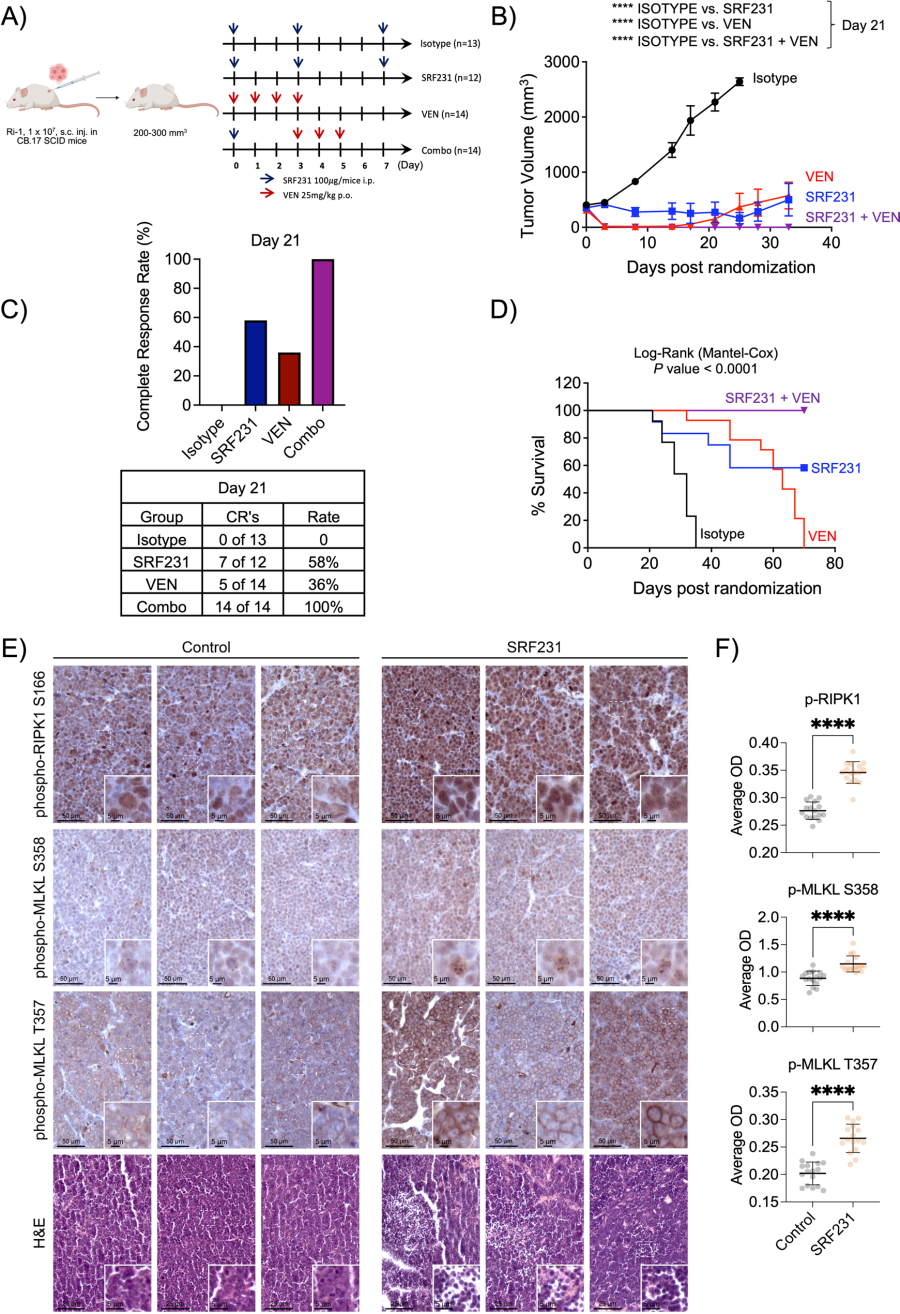
SRF231 is highly active in combination with venetoclax in a BCL-2 dependent DLBCL (Ri-1) xenograft model. **A.** Illustration of the 4 treatment regimens. Treatments were performed for one cycle. Created in BioRender. Chamberlain, S. (2026) https://BioRender.com/goslr3f. **B**. CB.17 SCID mice were implanted with Ri-1 tumor subcutaneously, randomized and treated with SRF231 (100 µg/mouse; Intraperitoneally, on days 0, 3, and 7), venetoclax (25 mg/kg; oral, days 0, 1, 2 and 3) or a combination of both (SRF231:100 µg/mouse on day 0 following three daily doses of venetoclax: 25 mg/kg on days 3, 4, and 5). Data are shown as mean tumor volumes ± SEM. (*P* < 0.0001 in all groups compared to hIgG4 isotype at day 21). Reported *P* values were calculated by Sidak’s multiple comparison test. Isotype hIgG4 (*n* = 13), SRF231 (*n* = 12), venetoclax (*n* = 14), SRF231 + venetoclax (*n* = 14). Results for specific treatment control groups of isotype, SRF231 alone, and venetoclax alone, with similar dosing schedules as combo group (third approach) could be found in supplemental Fig. S8B. **(C)** Bar graphs and tables showing the complete response (CR) rates and actual number of mice with CR, respectively, at day 21 for all treatment groups. **(D)** Kaplan-Meier plots of overall survival of Ri-1 tumor-bearing animals treated with SRF231 +/- venetoclax (Median survival: SRF231 vs. hIgG4 isotype: 32 days, venetoclax vs. hIgG4 isotype: 63 days). Statistics were calculated by Log-rank (Mantel-Cox) test. Results for specific treatment control groups of isotype, SRF231 alone, and venetoclax alone, with similar dosing schedules as combo group (third approach) could be found in supplemental Fig. S8D. **(E)** IHC staining of p-RIPK (S166), p-MLKL (T357 and S358) and H&E for hIgG4 control- or SRF231-treated Ri-1 tumors harvested 72 h from mice following one treatment of hIgG4 control or SRF231 (100 µg/mouse) (*n* = 3). **(F)** Quantification of 6 representative region of interests (ROI) per sample of p-RIP1K and p-MLKL IHC staining in Fig. 4E. (*n* = 3 per treatment). Reported *P* values were calculated by unpaired two-tailed Welch’s *t* test. OD: Optical Density

Based on our in vitro data for SRF231 and venetoclax on Ri-1 and primary CLL cells, we hypothesized that SRF231, venetoclax, and their combination would all provide substantial anti-tumor activity in vivo. We generated a xenograft model by implanting Ri-1 cells and we explored three different treatment approaches. Modeled after prior preclinical and clinical studies [18, 20], we designed the following 1-cycle treatment groups: (Approach 1) mice treated with isotype control or SRF231 on days 0, 3, and 7; (Approach 2) mice treated with venetoclax on days 0, 1, 2 and 3; (Approach 3) mice receiving a sequential, reduced-dosing combination regimen, with SRF231 administered on day 0, followed by venetoclax on days 3, 4, and 5 (Fig. 4A). The rationale for the sequential treatment and fewer doses in approach 3 was to ensure that SRF231 could target/bind a ‘live’ CD47 clustering on a non-compromised cell surface to drive its anti-tumor effect, as venetoclax treatment could potentially disrupt cell surface integrity due to membrane degradation from late apoptosis. These fewer doses in approach 3, if effective, could also reduce the risk of toxicity, both in the mouse model and eventually if studied in clinical trials. Approach 3 also includes treatment control groups of isotype, SRF231 alone, and venetoclax alone, with the exact dosing schedules and frequencies (Suppl. Figure S8B).

Upon establishing tumor masses, mice were randomized and treated with either hIgG4 isotype control, SRF231, venetoclax, or their combination (Fig. 4A). Both SRF231 and venetoclax displayed strong initial in vivo monotherapy activity in this lymphoma model; however, tumor relapse was observed in the monotherapy groups at later time points, despite having more doses being administered (Fig. 4B). In contrast, the group treated with the combination SRF231 and venetoclax exhibited sustained and complete tumor growth inhibition as compared to other approaches (Fig. 4B) or to its respective control groups (Suppl. Figure S8B). Similarly, the combination of SRF231 and venetoclax resulted in 100% complete response (CR) rate of animals treated, whereas SRF231 or venetoclax monotherapy resulted in lower CR rates of 33% and 50% at Day 17, 58% and 36% at Day 21, and 58% and 36% at Day 25, respectively (Fig. 4C, Suppl. Figure S8 C). The 100% CR rate of the SRF231 and venetoclax combination led to a clear and significant prolongation of survival in this in vivo model compared to hIgG4 isotype antibody control, SRF231 alone, and venetoclax alone (Fig. 4D, Suppl. Figure S8D). Notably, all mice from the combo group survived throughout the experiment (none reached humane endpoint). This in vivo observation was also re-capitulated in another BCL-2 dependent model of AML, HL60 (Suppl. Figure S9 A), where we observed that SRF231 could similarly induce p-MLKL and necroptosis in vitro (Suppl. Figure S9B, Suppl. Figure S3A) as well as SRF231 and venetoclax combination could completely inhibit tumor growth and prolonged survival in vivo without any mouse reaching humane endpoint (Suppl. Figure S9 C-S9E).

Finally, we performed IHC staining of p-RIPK1 and p-MLKL on the tumors of hIgG control- or SRF231-treated Ri-1 xenograft mouse model to assess for induction of necroptosis. Indeed, we observed a significant increase in p-RIPK1 and two different p-MLKL (T357, S358) residues in the SRF231-treated samples as compared to its hIgG4 controls (Fig. 4E and F, staining controls displayed in Suppl. Figure S8 A). Corroborating that, H&E staining further showed extensive necrotic regions in tumors treated with SRF231 (Fig. 4E), thus collectively confirming that SRF231 induces necroptosis in these tumors.

### SRF231 overcomes venetoclax resistance in models of lymphoid malignancies

**Fig. 5:**
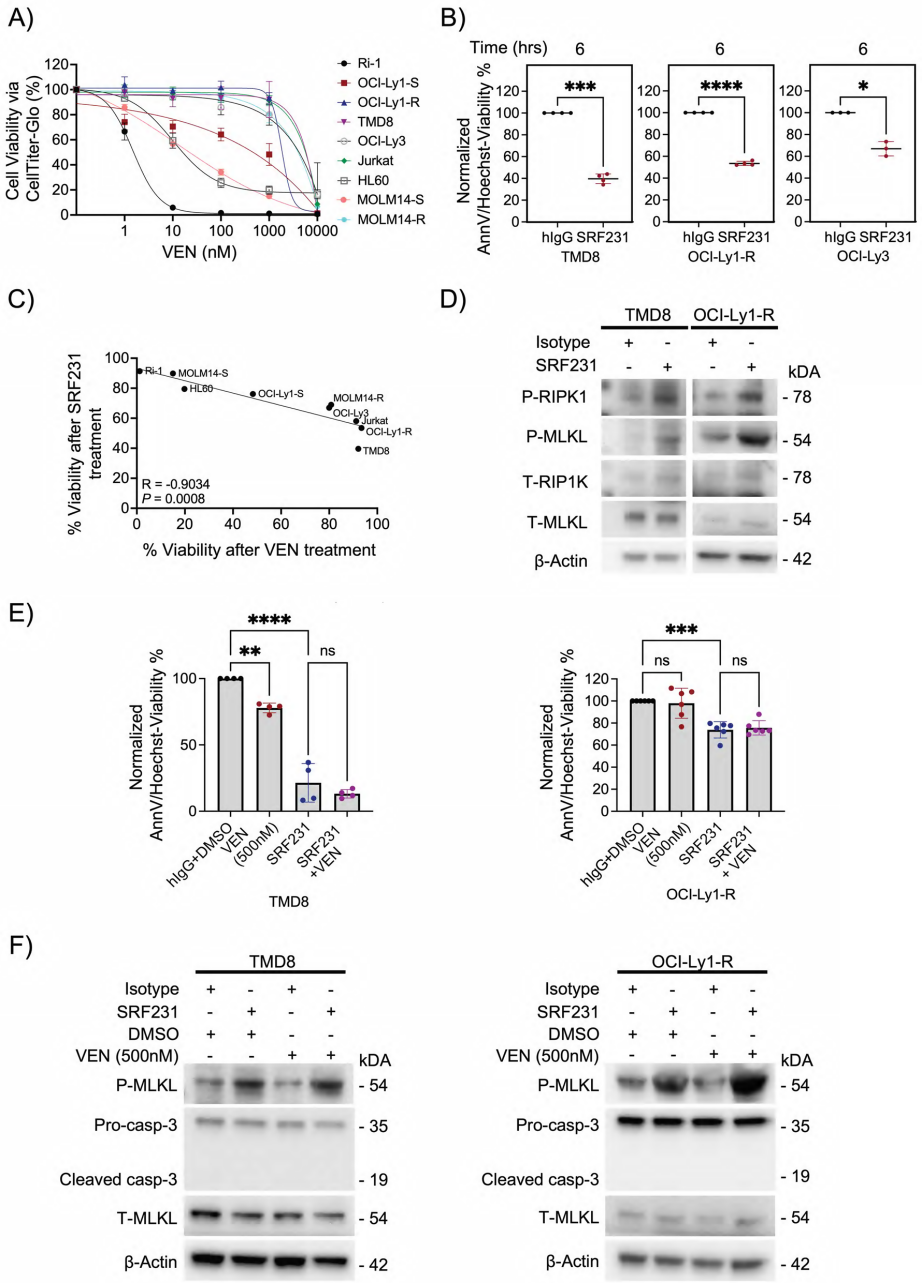
SRF231 alone is active in lymphoid malignant cells resistant to BCL-2 inhibition. **(A)** Cell viability of 9 cancer cell lines was measured via CTG assay following treatment with increasing doses of venetoclax for 48 h. **(B)** Cell death inductions of TMD8 (*n* = 4), OCI-Ly1-R (*n* = 4) and OCI-Ly3 (*n* = 3) cells were measured following Protein G-bound SRF231 incubation with AnnV/Hoechst assay for 6 h. Reported *P* values were calculated by paired Student’s *t* test. **(C)** Pearson correlation coefficient analyses between % viability of cells treated with 10 µg/ml SRF231 for 6 h and % viability of cells treated with 1 µM venetoclax for 48 h. **(D)** Western blots showing the increased p-RIPK1 (S166) and p-MLKL (S358) after Protein G-bound SRF231 treatment of TMD8 and OCI-Ly1-R cells (10 µg/ml, 6 h). **(E)** Cell viability was measured via AnnV/Hoechst assay for TMD8 and OCI-Ly1-R cells following concurrent treatment with protein G-bound SRF231 (SRF231:10 µg/ml) and venetoclax (500nM) for 6 h. (TMD8, *n* = 4; OCI-Ly1-R, *n* = 6). Reported *P* values were calculated by Sidak’s multiple comparison test. **(F)** Western blots showing the increased p-MLKL (S358) but not caspase 3 cleavage following treatment with Protein G-bound SRF231 or venetoclax or both in TMD8 and OCI-Ly1-R cells

Recent studies have demonstrated that venetoclax resistant cells display consistent mechanisms of apoptotic pathway evasion [25, 27, 29, 67, 68]. To decipher whether SRF231 could overcome venetoclax resistance, we screened and selected 5 lymphoid and myeloid cell lines relatively resistant to venetoclax – inherently resistant Jurkat (BAX-deficient), OCI-Ly3, TMD8 (reduced mitochondrial priming) and acquired resistant OCI-Ly1-R, MOLM14-R (functionally dependent on MCL-1) (Fig. 5A) [25, 27, 29]. Following 6-hour SRF231 treatment, we found that SRF231 could induce cell death more efficiently in the resistant cell lines compared to BCL-2-dependent Ri-1, OCI-Ly1-S, MOLM14-S and HL60 at 6 h (Figs. 1I and 5B, Suppl. Figure S10 A), thus suggesting a particular vulnerability to necroptosis in these cells that are resistant to apoptosis. This is further corroborated by our Pearson correlation coefficient analysis showing a strong and significant negative correlation between % viability of cells following SRF231 and venetoclax treatment (*R* = −0.9034, *P* = 0.0008), thus suggesting that cells that are less susceptible to venetoclax, are more susceptible to SRF231 (Fig. 5C). In addition, we observed that SRF231 could consistently induce the increase in p-RIP1K and p-MLKL, thus confirming necroptosis activation in these resistant cells (Fig. 5D, Suppl. Figure S10B).

We next investigated whether venetoclax could potentiate the cytotoxic effect of SRF231 towards these resistant cells. Consistent with our hypothesis, SRF231 did not re-sensitize these cells to venetoclax, and the combination of SRF231 and venetoclax did not further enhance cell death, even at a higher venetoclax dose of 500nM (Fig. 5E). Western blotting further showed that necroptosis was activated (increased p-MLKL expression), but apoptosis was not (no cleaved caspase-3 generated) (Fig. 5F). These data collectively suggest that SRF231 alone is sufficient to target apoptosis-resistant cells, and a translational implication to be explored is that in patients resistant to venetoclax, discontinuation of venetoclax and switching to CD47 blockade alone may be a viable therapeutic strategy.

**Fig. 6:**
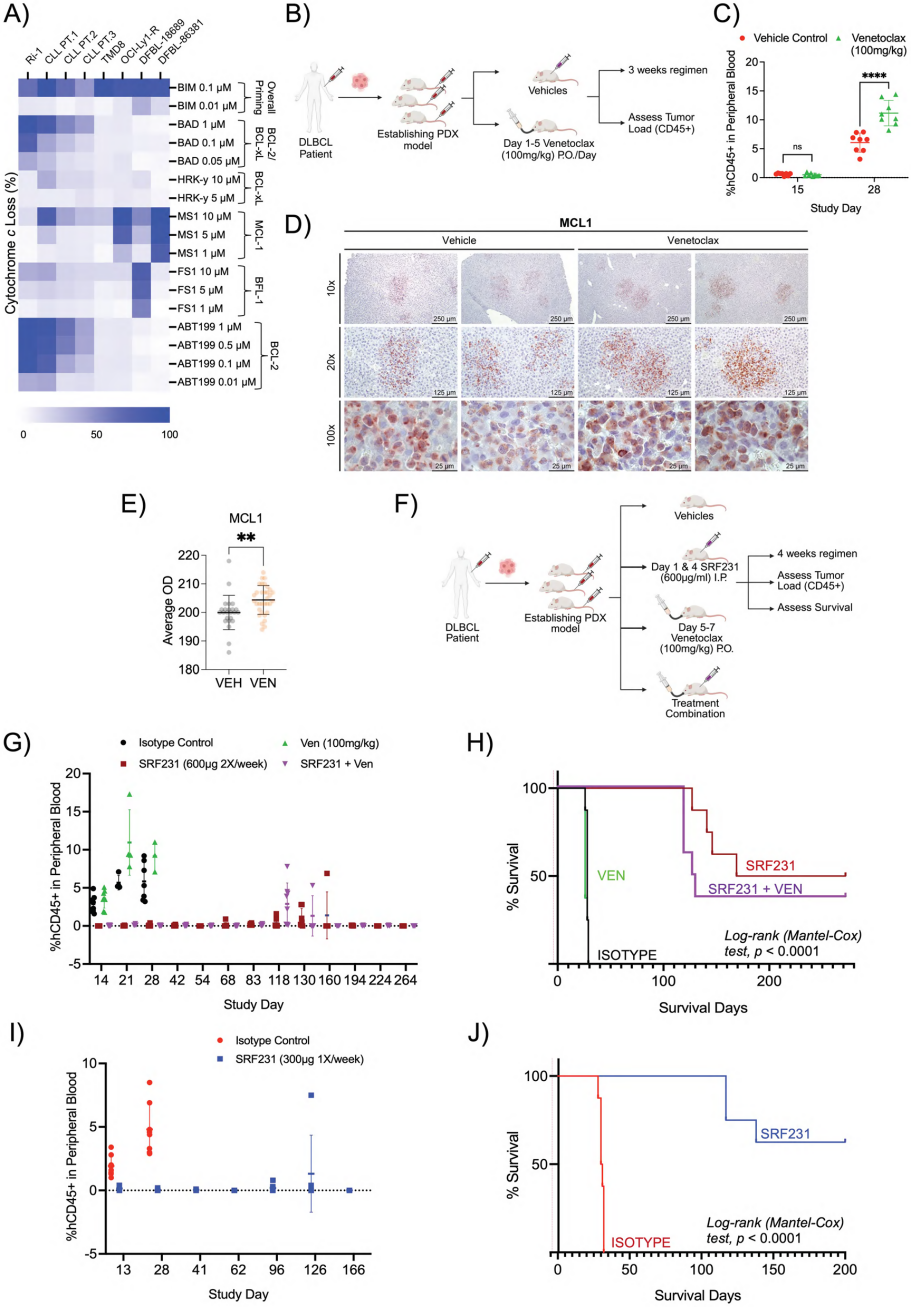
SRF231 alone is highly active in venetoclax resistant patient derived DLBCL xenograft model. **(A)** BH3 profiling displays anti-apoptotic dependences of lymphoid malignant Ri-1, primary CLL cells from 3 individual patients, TMD8, OCI-Ly1-R and primary DLBCL cells from 2 patients. Right side labels indicating specific anti-apoptotic protein dependences. **(B)** Illustration of the establishment of DLBCL PDX model with treatment regimen and tumor load measurement by hCD45 + via flow cytometry. Created in BioRender. Chamberlain, S. (2026) https://BioRender.com/2pm3alv. **(C)** Tumor load measurement via hCD45 + of mice peripheral blood following treatment with vehicle control or venetoclax (100 mg/kg P.O.) at day 15 and 28. Reported *P* values were calculated by Sidak’s multiple comparison test. **(D)** IHC staining of hMCL-1 from the liver of vehicle control- or venetoclax-treated mice. Liver harvested a week after first round of venetoclax treatment (*n* = 3). **(E)** Quantification of 6 representative region of interests (ROI) per sample of hMCL-1 IHC staining in Fig. 6D. (*n* = 3 per treatment). Reported *P* values were calculated by unpaired two-tailed *t* test. OD: Optical Density. **(F)** Illustration of the establishment of DLBCL PDX model with 4-arm treatment regimen, tumor load measurement by hCD45 + via flow cytometry and survival analysis. Created in BioRender. Chamberlain, S. (2026) https://BioRender.com/7ev2abw. **(G)** Tumor load measurement via hCD45 + of mice peripheral blood following treatment with hIgG4 control, venetoclax (P.O.), SRF231 (I.P) and combo for the indicated days. **(H)** Kaplan-Meier plots of overall survival of DLBCL tumor-bearing PDX animals treated with SRF231 +/- venetoclax (*n* = 8). Statistics were calculated by Log-rank (Mantel-Cox) test. **(I)** Tumor load measurement via hCD45 + of mice peripheral blood following treatment with hIgG4 control and SRF231 (I.P) for the indicated days. **(J)** Kaplan-Meier plots of overall survival of DLBCL tumor-bearing PDX animals treated with hIgG4 control or SRF231 (*n* = 8). Statistics were calculated by Log-rank (Mantel-Cox) test

To further validate our in vitro finding that SRF231 effectively targets venetoclax resistant cells, we utilized BH3 profiling to study primary samples from patients with CLL and DLBCL to identify those that had reduced BCL-2 dependence (Fig. 6A). Overall, we observed that primary DLBCL patient samples (DFBL-18689 and DLBL-86381), DLBCL cell lines (TMD8 and OCI-Ly1-R) and certain primary CLL patient sample (CLL PT.3) have relatively reduced BCL-2 dependence.

We then generated a PDX mouse model from the DLBCL patient sample (DFBL-18689) with reduced BCL-2 dependence, and subsequently treated the mice with venetoclax on a 5-day course per week for 3 weeks to ascertain susceptibility to venetoclax (Fig. 6B). We found that venetoclax not only failed to reduce tumor burden of these DLBCL-PDX mice, confirming venetoclax resistance, but also led to an increased in tumor burden (Fig. 6C), thus suggesting a potential drug addiction. The latter was further supported by the stabilization of MCL-1 anti-apoptotic protein, a previously reported association in samples from patients who have developed resistance to venetoclax [29] (Fig. 6D and E).

Given that these primary DLBCL cells are not responsive to venetoclax, we proceeded to test these conditions in the PDX model – isotype control, SRF231, venetoclax and combination treatment (Fig. 6F), in order to validate our in vitro observations (Fig. 5E). To reduce the risk of drug addition, we lowered the frequency of venetoclax to a 3-day course per week for 4 weeks. SRF231 alone and the SRF231 and venetoclax combination both showed complete suppression of tumor burden, as compared to vehicle and venetoclax alone (Fig. 6G). Consistent with our prior findings, venetoclax alone again showed increased tumor burden as compared to vehicle, suggesting that even with a lower frequency of venetoclax, these resistant cells could still gain a proliferative advantage (Fig. 6G).

Interestingly, we observed a more rapid relapse in the combination arm as compared to SRF231 alone at day 118 (Fig. 6G). We also found that mice from the SRF231 and venetoclax combination arm succumbed to disease relapse at a faster and higher rate than those from the SRF231 monotherapy arm (Fig. 6H). These observations validate our hypothesis that venetoclax is not a suitable partner for SRF231 in an apoptotic-resistant or non-BCL-2 dependent setting. Given the effectiveness of SRF231 monotherapy in suppressing tumor growth in this resistant model, we finally proceeded to halve both its dose and frequency, as an attempt to mitigate potential toxicity. Intriguingly, we observed that SRF231 at 300 µg administered once per week could still effectively suppress tumor growth and prolong mice survival at a similar rate (mice survival: 62.5%) compared to its original dose and frequency (50%) (Fig. 6H and J). Collectively, our work demonstrates that SRF231 alone is highly active in models of apoptosis and venetoclax resistance.

## Discussion

We found that targeting CD47, a macrophage checkpoint molecule, leads to anti-tumor activity via the induction of tumor cell phagocytosis and non-apoptotic cell death, providing an alternative therapeutic approach to adaptive immune checkpoint blockade strategies such as PD-1 and CTLA-4 inhibitors. To maximize the benefits of CD47 blockade in lymphoid malignancies, it is crucial to unravel the cell death mechanism to rationally identify optimal combination partners. Here, we demonstrated that SRF231 could trigger necroptosis in vitro in a panel of lymphoid and myeloid malignant cell lines and primary CLL patient samples, and in vivo in cell line- and patient-derived xenograft models. The new mechanistic insights demonstrate that SRF231 activates both phagocytotic and necroptotic pathways, thereby also potentially sensitizing cells to drugs that induce apoptosis. Thus, our study suggests a promising combination approach by targeting multiple complementary cell death modalities (phagocytosis, necroptosis and apoptosis) and justifies exploring such combinations in the clinic for patients with lymphoid malignancies.

We also demonstrated that CD47 blockade in combination with venetoclax is unlikely to be effective in lymphoid malignant cells that are resistant to BCL-2 inhibition. Importantly, we found that in the setting of venetoclax resistance, continuing venetoclax may be counter-productive, as it may further stabilize the anti-apoptotic protein MCL-1 and promote tumor growth. Our work therefore highlights the need to assess whether patients’ tumor cells are functionally resistant to BCL-2 inhibition with techniques such as BH3 profiling, and if that is the case, to explore switching to CD47 blockade alone rather than combining with a BCL-2 inhibitor. Given that clinical trials of CD47 blockade may include venetoclax, it is therefore necessary to identify and stratify suitable patients for CD47 blockade mono- or combination therapy.

Most drugs utilized as anti-cancer therapy, including traditional cytotoxic chemotherapy and targeted small molecule inhibitors, kill tumor cells through apoptosis. Although these anti-cancer drugs are highly effective in inducing apoptosis, certain population of cancer cells within the tumor may escape apoptosis due to cancer cell heterogeneity or spatial differences that prevents drug exposure. As such, orthogonal therapeutic approaches to improve tumor cell death by targeting these particular cancer cell populations may have broad clinical applicability. Inducing necroptosis represents one such promising approach. Disrupting the CD47/SIRPα pathway through CD47 blockade therapy in combination with rituximab has shown a high level of clinical activity and good tolerability in lymphoid malignancies [69, 70]. Our study further identified venetoclax as a new partner to explore in combination with anti-CD47 antibodies, which is a promising approach to explore therapeutically for lymphoid malignancies that are not resistant to BCL-2 inhibition as a way to further improve the depth of response and duration of remission. We used the functional precision medicine BH3 profiling technique to identify the BCL-2 inhibitor venetoclax as a promising partner for SRF231 in malignant lymphoid cells that depend on BCL-2 for survival, such as CLL cells [27], though our findings are likely to be applicable also to other drugs that selectively inhibit these targets.

In light of new resistance mechanisms recently identified for venetoclax, such as BCL-2 family gene mutations, BCL-2 family protein hyperphosphorylation, and increased reactive oxygen species levels, which impair apoptotic induction [23–27, 29, 71, 72], it is important to continue to search for alternative approaches to tackle refractory diseases, particularly when the apoptotic pathway is not functional, leading to resistance to BCL-2 inhibition. Indeed, in our cell lines of DLBCL that are not dependent on BCL-2, venetoclax was not only ineffective but also appeared to drive tumor growth in our DLBCL PDX model. This is likely due to stabilization of the anti-apoptotic MCL-1 protein, a phenomenon previously reported in patients with CLL, DLBCL, or AML who become refractory to venetoclax [25, 29, 73]. In such cases of apoptotic resistance, SRF231 monotherapy may still be active, given its ability to activate both phagocytosis and necroptosis.

Our results are also consistent with other studies that demonstrate that CD47 blockade may induce caspase-independent programmed cell death [47, 48]. We anticipated that our data would not be specific to SRF231, but rather would apply broadly to anti-CD47 therapies. This was seen in both magrolimab and B6H12. Although a rather modest increase in p-MLKL was observed with magrolimab, necroptotic-cell death could still be induced. Similarly, B6H12 at a higher concentration was also seen to induce p-MLKL and necroptotic-cell death. Our data therefore demonstrate that CD47 antibodies in general could induce p-MLKL-dependent necroptosis, albeit at variable degrees and concentrations. This could potentially be due to the design of the antibodies, as magrolimab also has specific toxicities such as RBC agglutination leading to anemia [10, 13, 64, 65], a concern that was absent with SRF231 and other newer CD47 mAbs [18, 74]. While recent clinical trials of magrolimab with azacitidine +/- venetoclax in AML were not successful, our work suggests that there may be important mechanistic differences between the drugs that target CD47, and that it is still worthwhile to pursue further study of targeting this pathway therapeutically, especially for cases that are resistant to BCL-2 inhibition.

Our observation that venetoclax treatment in combination with SRF231 was not effective in clearing tumor burden but rather accelerated relapse in models of venetoclax and apoptotic resistance seems at first glance to be counterintuitive. However, we noted that mice treated with venetoclax had increased MCL-1 levels, which may resist the effects of necroptosis and explain the observed faster relapse and shorter survival in such mice. Supporting this hypothesis, disruption of MCL-1 expression has been linked to necroptosis initiation [75]. Another study also showed that MCL-1 inhibition displayed necrosis induction [76]. Moreover, MCL-1 at the mitochondrial matrix is necessary to facilitate normal mitochondrial fusion as well as possesses the ability to inhibit mitochondrial fission factor (Mff)-mediated mitochondrial fragmentation [77, 78]. Given that mitochondrial fission or fragmentation is required for necroptosis [58], the increase in MCL-1 may serve as an inhibitory mechanism towards necroptosis. Supporting this, it was reported that MLKL translocation to the mitochondria following necroptosis induction was also accompanied by the reduction of MCL-1 at the mitochondrial matrix [75]. These findings thus strongly suggest that upregulated MCL-1 could play a restrictive role on SRF231/CD47 blockade-induced necroptosis and that venetoclax should not be used with SRF231 in settings where cancer cells do not depend on BCL-2 for survival. Our findings also suggest that SRF231 could be more effective in eliminating cancer cells that are resistant to venetoclax, provided that these cells are not simultaneously treated with venetoclax in a scenario where venetoclax does not induce any cytotoxic effect but rather oncogenic effect through MCL-1 upregulation.

Other agents that directly stimulate necroptosis have also been identified. For example, shikonin, the first reported small molecule to induce necroptosis, was found to bypass BCL-2- and BCL-XL-mediated apoptotic resistance [79]. Recently, several compounds such as SMAC mimetics, anti-metabolites like fluorouracil, and bortezomib have been reported to trigger necroptosis in cancer cells, suggesting a growing recognition of the potential of this approach [53, 80]. Additionally, despite some recent setbacks, other CD47 checkpoint blockade drugs are still being studied in the clinic, including a humanized CD47 antibody (NCT06196203), bispecific antibodies (NCT03804996, NCT04881045), a CD47 fusion protein (NCT04417517) and several others [81]. Interestingly, strategies with SIRPα-blockade with antibodies to induce phagocytosis of cancer cells are also now being tested. Future studies could explore the ability of these agents to induce necroptosis through CD47 blockade.

Finally, though our findings are robust, our study does have limitations to highlight. Given that CD47 antibodies were scaffolded on the G-coated protein plates through incubating them on the G-coated protein plate overnight, there may be slight variability in terms of the amount of antibodies being scaffolded on the G-coated protein plate. Another limitation to be taken into account is the amount of G-coated proteins available on the plate to allow for antibodies to be bound. These may collectively determine the overall necroptotic effects of scaffolded CD47 antibodies on the cancer cells. Hence our results could likely be further enhanced and corroborated in the future if these subtle but potentially critical factors could be quality-controlled through standardization of a fixed amount of scaffolded antibodies on the G-coated protein plates. Nonetheless, our overall data and message still point to the fact that CD47 blockade could consistently induce MLKL-dependent necroptotic-cell death across a broad array of cancer cell lines and xenograft models.

## Conclusion

In summary, our work deepens the understanding of the biology and mechanisms of CD47 blockade to help inform the exploration of novel therapeutic approaches utilizing rationally-identified complementary drugs. To further refine this approach, we demonstrated how utilizing the functional precision medicine technique BH3 profiling may be useful to assess for sensitivity to BCL-2 inhibition, which would inform whether to use CD47 blockade alone or in combination with venetoclax. These insights will help us to optimize therapeutic outcomes for patients with a broad range of lymphoid malignancies as we work to develop novel therapeutic strategies utilizing CD47 blockade in the clinic.

## Supplementary Information

Supplementary Material 1

## Data Availability

All data and materials are available upon reasonable request from the corresponding author.

## Abbreviations

AML: Acute myeloid leukemia
AnnV: Annexin V
BCL-2: B-cell leukemia/lymphoma-2
BH3: BCL-2 homology-3
BTK: Bruton tyrosine kinase
CLL: Chronic lymphocytic leukemia
CR: Complete response
CTLA-4: Cytotoxic T-lymphocyte-associated protein-4
DLBCL: Diffuse large B-cell lymphoma
H&E: Hematoxylin and Eosin
hMDMs: Human monocyte-derived macrophages
IHC: Immunohistochemistry
IP: Intraperitoneal
KO: Knockout
mAb: Monoclonal antibody
MLKL: Mixed-lineage kinase domain–like pseudokinase
NEC-1: Necrostatin-1
NHLs: Non-Hodgkin lymphomas
PBMCs: Peripheral blood mononuclear cells
PD-1: Programmed death-1
PD-L1: PD-1 ligand
PDX: Patient-derived xenograft
p-MLKL: phospho-MLKL
p-RIPK1: phospho-RIPK1
ProXe: Public Repository of Xenografts
RBC: Red blood cell
RIPK1/RIPK3: Receptor-interacting protein kinases 1/3
ROIs: Region of interests
VEN: Venetoclax
WT: Wild-type
