# Supplementary material for: CD47 blockade-driven necroptosis complements BCL-2 inhibition-driven apoptosis in lymphoid malignancies
Source: J Hematol Oncol. 2026 Jan 3;19:11. doi: 10.1186/s13045-025-01774-3 (PMC12810019; doi:10.1186/s13045-025-01774-3)
Supplement: Supplementary file 1 — Supplementary Material 1 [file 13045_2025_1774_MOESM1_ESM.pdf]

Supplemental Figure 1

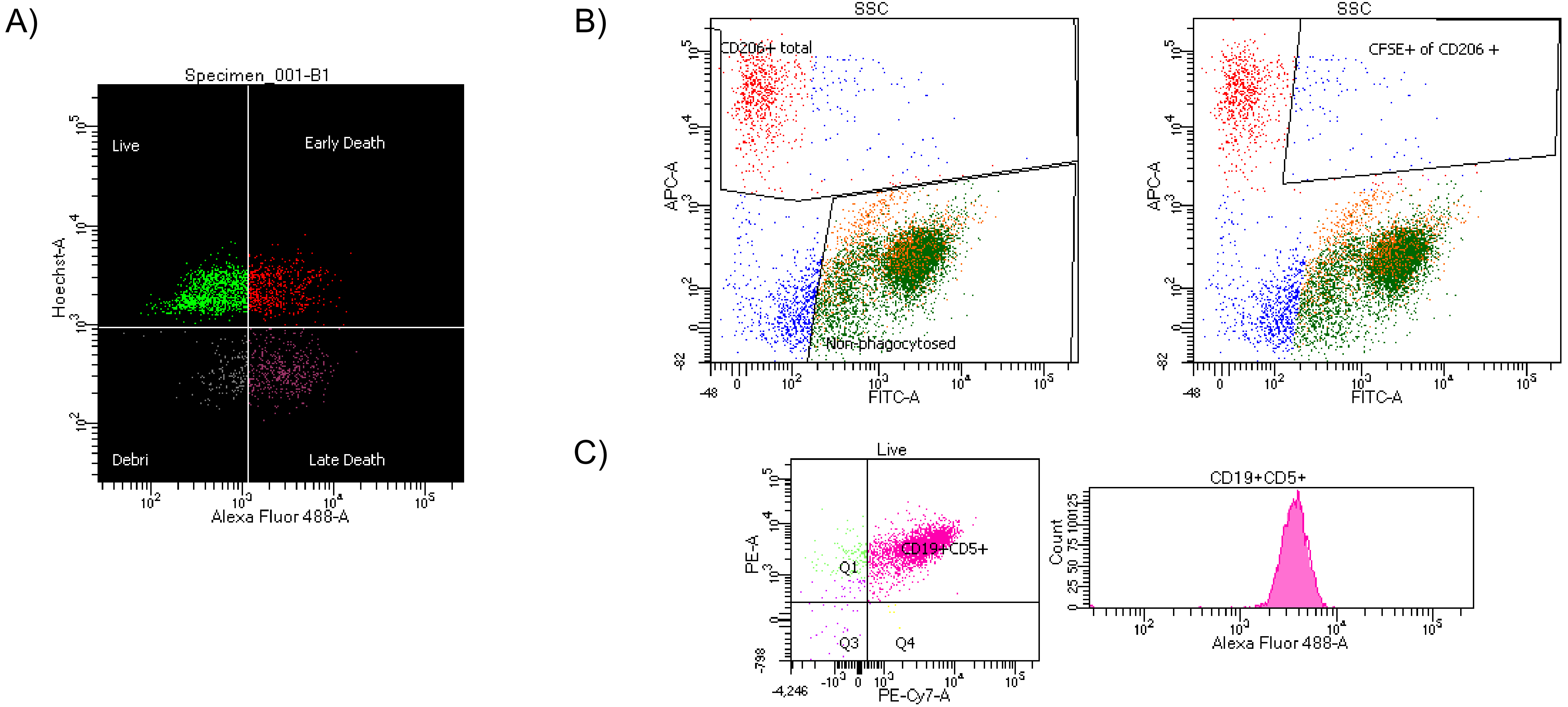

Supplementary Figure 1. Gating strategies for Annexin V (AnnV)/Hoechst, phagocytosis and BH3 profiling assays.

(A) Panel shows live cells depicted at top left quadrant, cells undergoing early death depicted at top right quadrant, debris depicted at lower left quadrant and cells undergoing late death depicted at lower right quadrant.

(B) Left panel shows gating of CD206+ macrophages and gating of non-phagocytosed CFSE+ cells. Right panel shows gating of CD206+ and CFSE+ double positive cells phagocytosed by macrophages.

(C) Left panel shows the gating of CD19+ and CD5+ double positive primary CLL cells and right panel shows the Alexa Fluor 488-cytochrome *c* signal of CD19+ and CD5+ gated cells.

Supplemental Figure 2

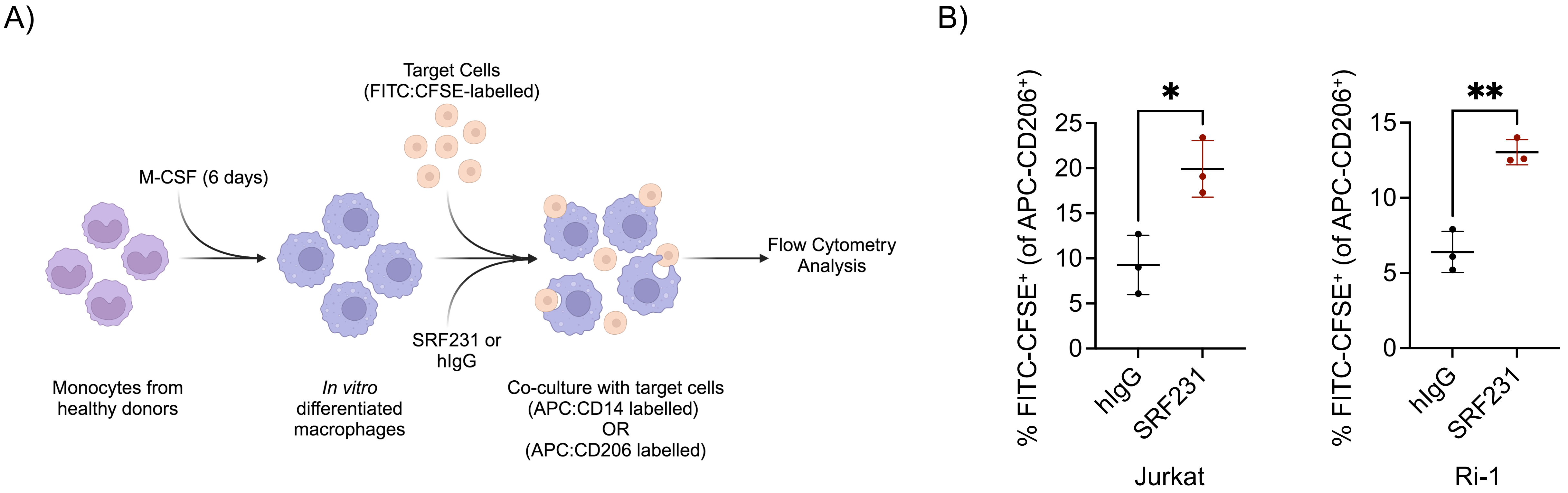

Supplementary Figure 2. Treatment with SRF231 induces canonical phagocytosis.

(A) Schematic illustration of the phagocytosis assay. Primary monocytes from healthy donors were cultured for 6 days in the presence of 100 ng/mL hM-CSF (Life Technologies) for in vitro macrophage differentiation (hMDMs), subsequently resuspended in flat-bottomed 96 well plates and left overnight before phagocytosis assay was performed. hMDMs were then cocultured with CFSE-labeled target cells and exposed to CD47 mAbs or isotype control. To track the degree of phagocytosis, macrophages were then stained with fluorescently-labeled anti-CD14 or anti-CD206 and the percentage of CD14:CFSE or CD206:CSFE double-positive cells were determined. Phagocytosis is identified as the $CD14^+$ or $CD206^+$ population with uptake of CFSE positive lymphocytes or cancer cells. Created in BioRender. Chamberlain, S. (2026) https://BioRender.com/2vzc0uh.

(B) Flow data indicating phagocytosis, determined from CD206:CSFE double-positive cells, following 2-hour treatment with either hIgG4 control or SRF231 at 10µg/ml. Reported *P* values were calculated by paired Student’s *t* test.

Supplemental Figure 3

A)

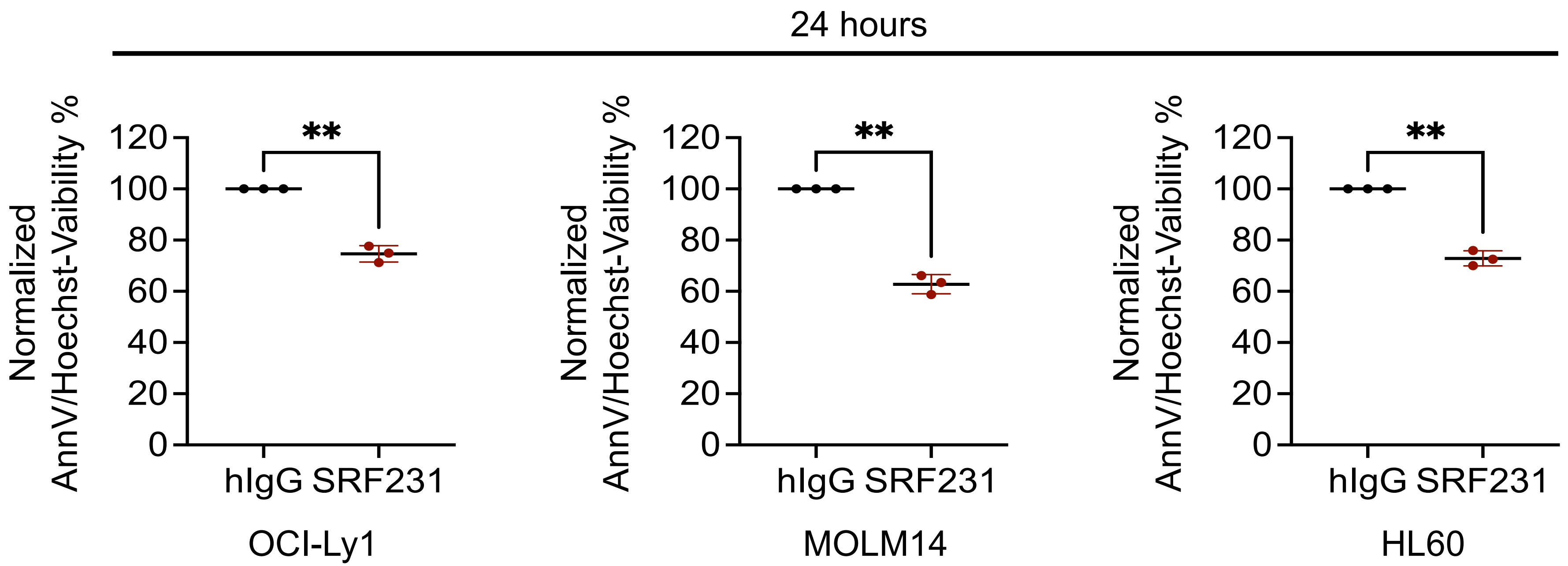

Supplementary Figure 3. Treatment with SRF231 induces cell death in various hematologic malignant cells.

(A) Cell death inductions of OCI-Ly1 (n=3, DLBCL), MOLM14 (n=3, AML) and HL60 (n=3, AML) cells were measured with AnnV/Hoechst assay following 24-hour Protein G-bound SRF231 incubation. Reported *P* values were calculated by paired Student's *t* test.

Supplemental Figure 4

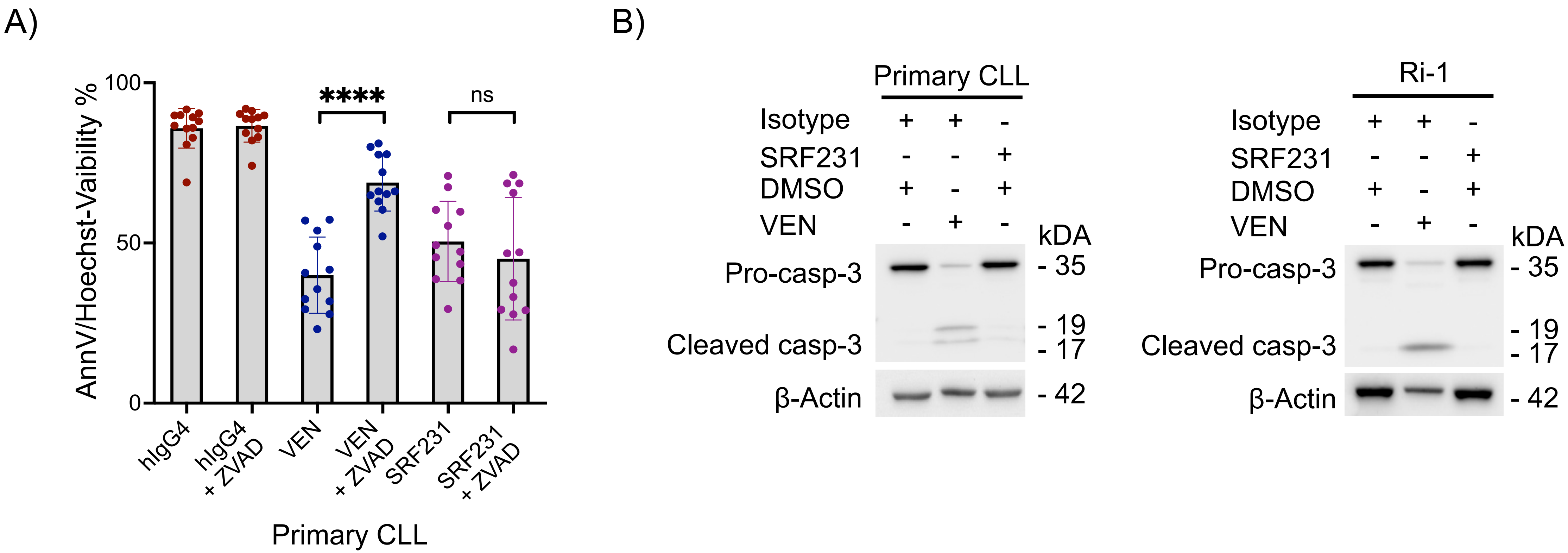

Supplementary Figure 4. SRF231 does not induce cell death by apoptosis.

(A) Cell viability was measured via AnnV/Hoechst assay for 12 primary CLL cells pre-treated with 50 μM pan-caspase inhibitor (Z-VAD-FMK) for 30 minutes prior to treatment with protein G-bound SRF231 for 6 hours. Venetoclax (VEN, 25nM) was used as positive control for apoptosis. Reported *P* values were calculated by Sidak's multiple comparison test.

(B) Western Blot showing the absence of caspase 3 cleavage following Protein G-bound SRF231 treatment of primary CLL cells (10 μg/ml, 6 hours) or Ri-1 cells (10 μg/ml, 24 hours). Venetoclax (25nM) was used as positive control for caspase 3 cleavage.

Supplemental Figure 5

A)

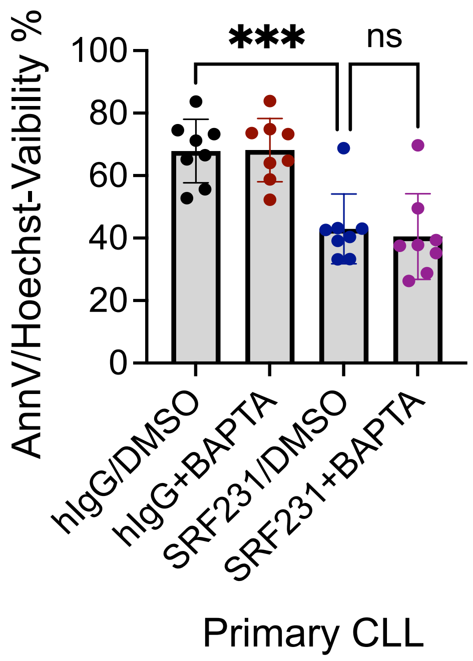

B)

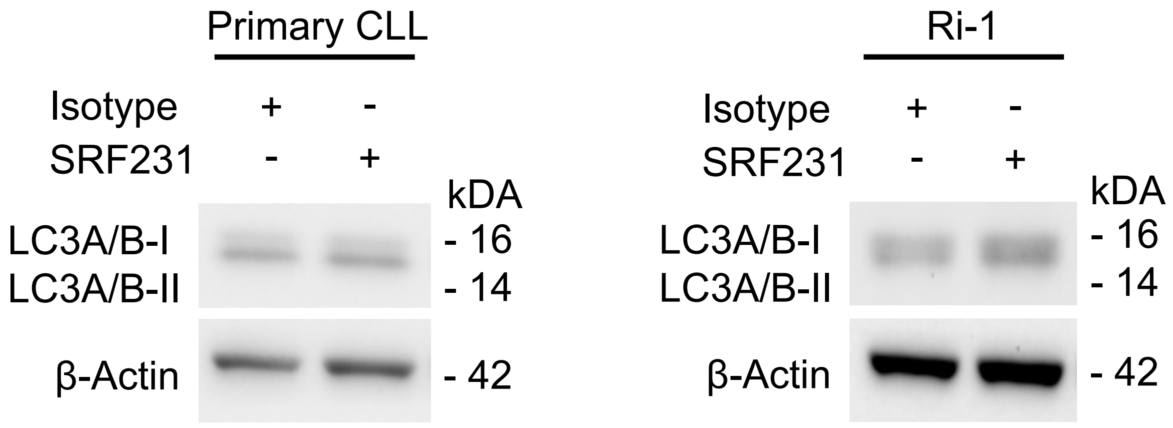

Supplementary Figure 5. SRF231 does not induce cell death by autophagy.

(A) Cell viability was measured via AnnV/Hoechst assay for 8 primary CLL cells pre-treated with 0.25 µM BAPTA for 30 minutes prior to treatment with protein G-bound SRF231 for 6 hours. Reported *P* value was calculated by Sidak's multiple comparison test.

(B) Western Blot showing the unaltered levels of LC3A/B-I and LC3A/B-II following Protein G-bound SRF231 treatment of primary CLL cells (10 µg/ml, 6 hours) or Ri-1 cells (10 µg/ml, 24 hours).

Supplemental Figure 6

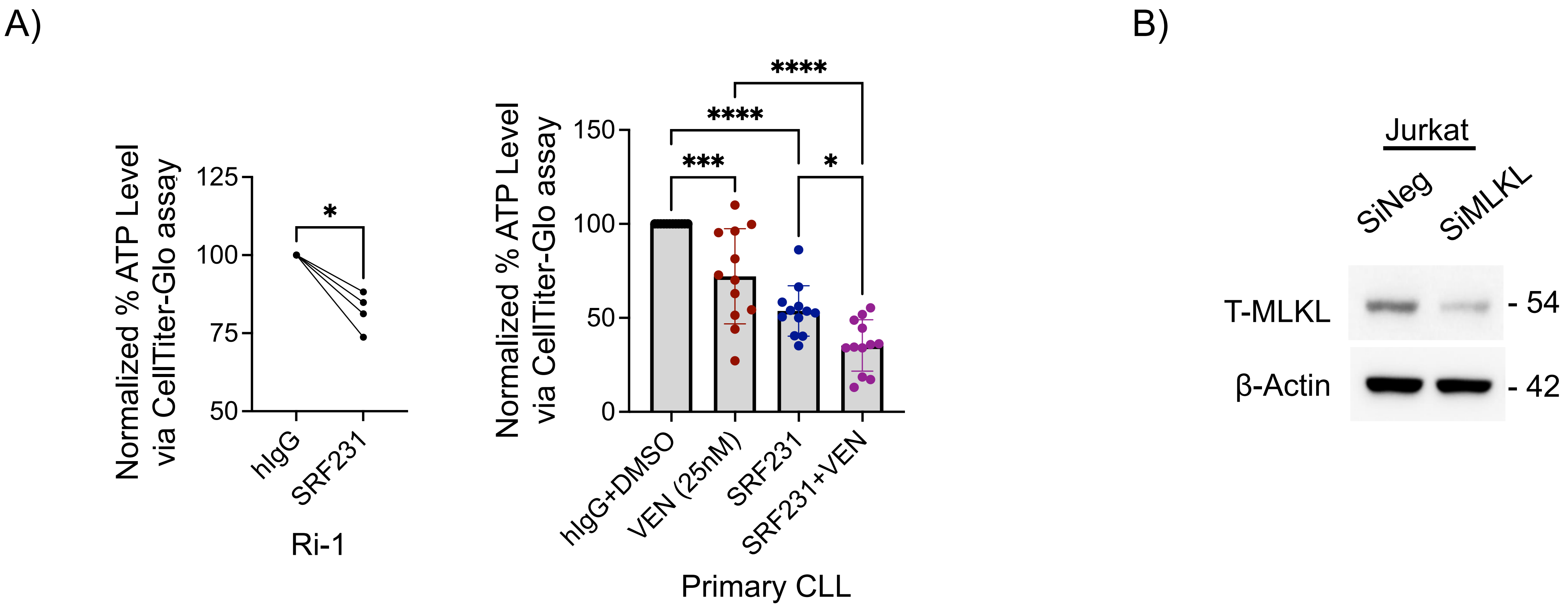

Supplementary Figure 6. CD47 blockade induces cell death and may involve MLKL protein.

(A) % ATP levels of Ri-1 and primary CLL cells measured by CTG assay following treatment with hIgG4 control, SRF231, venetoclax or combo. Reported *P* values were calculated by paired Student's *t* test (Ri-1) or Sidak's multiple comparison test (CLL).

(B) Western blot analysis showing the knockdown of total MLKL protein expression following MLKL siRNA treatment (50nM, 48 hours) on Jurkat cells.

Supplemental Figure 7

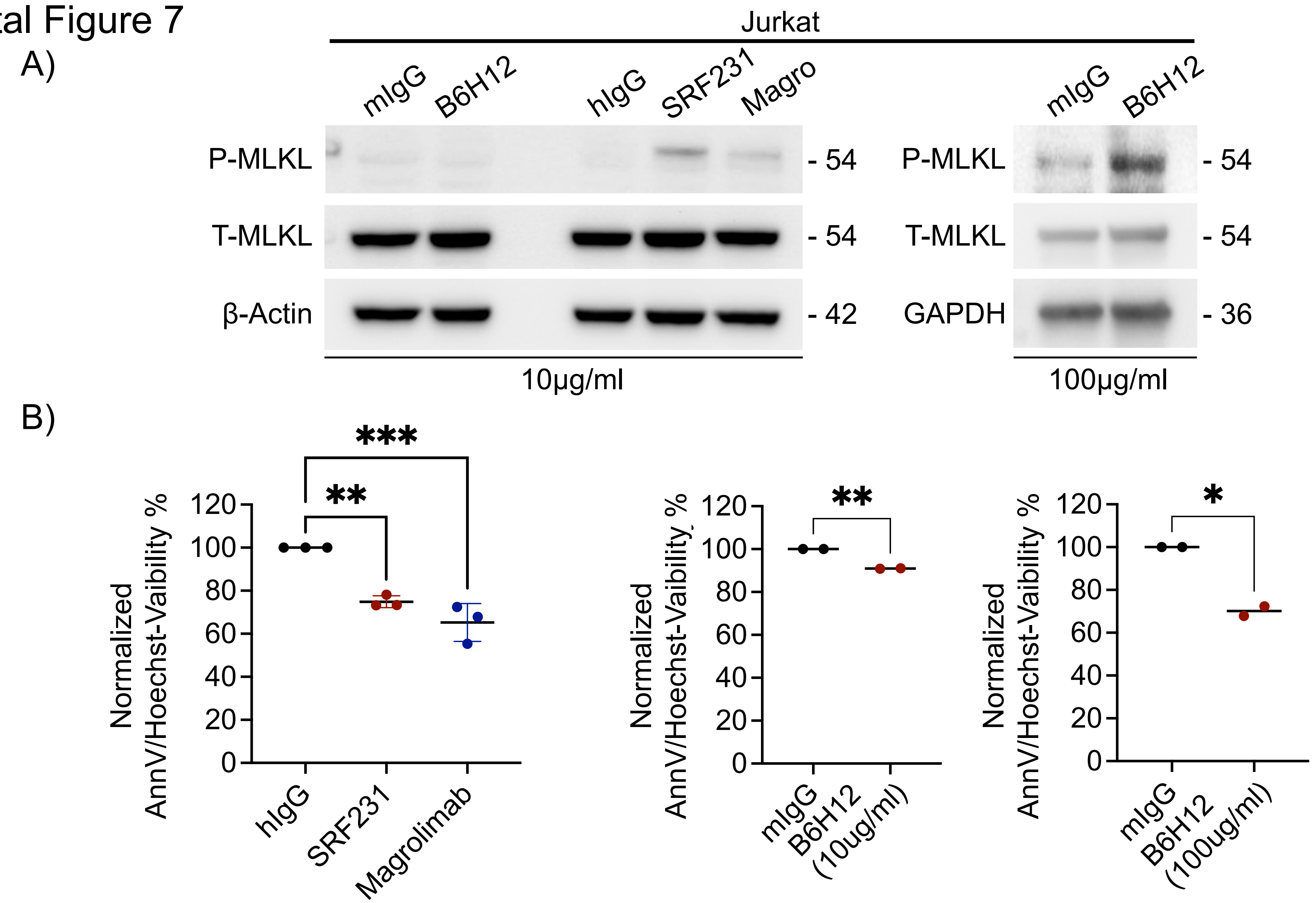

Supplementary Figure 7. CD47 antibodies generally induce necroptotic-cell death.

(A) Western Blot showing p-MLKL following treatment with Protein G-bound m/hIgG, SRF231, Magrolimab (10μg/ml, 24 hours) or B6H12 (10, 100μg/ml, 24 hours) of Jurkat cells.

(B) Cell death inductions of Jurkat cells were measured with AnnV/Hoechst assay following 24-hour Protein G-bound SRF231, magrolimab (10μg/ml, 24 hours) or B6H12 (10, 100μg/ml, 24 hours) incubation. Reported *P* values were calculated by either Sidak's multiple comparison test or paired Student's *t* test (B6H12).

Supplemental Figure 8

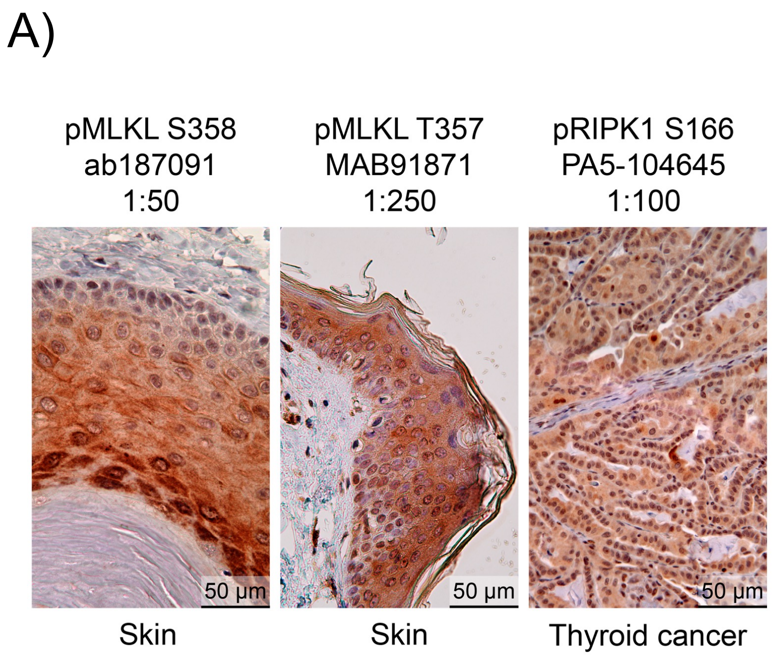

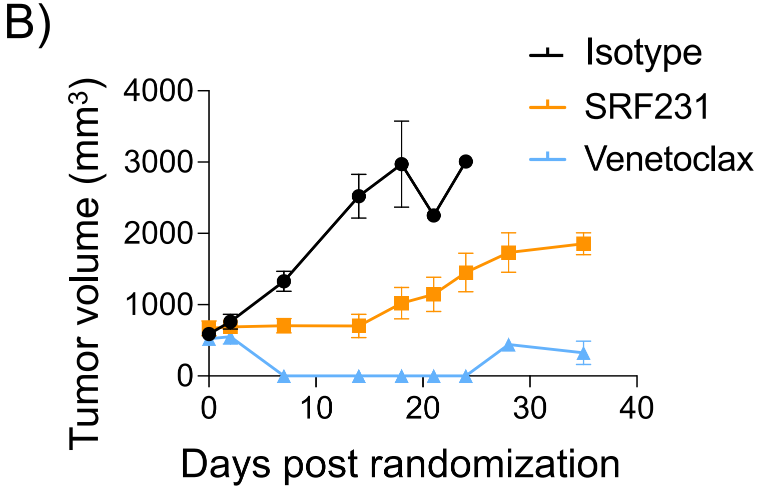

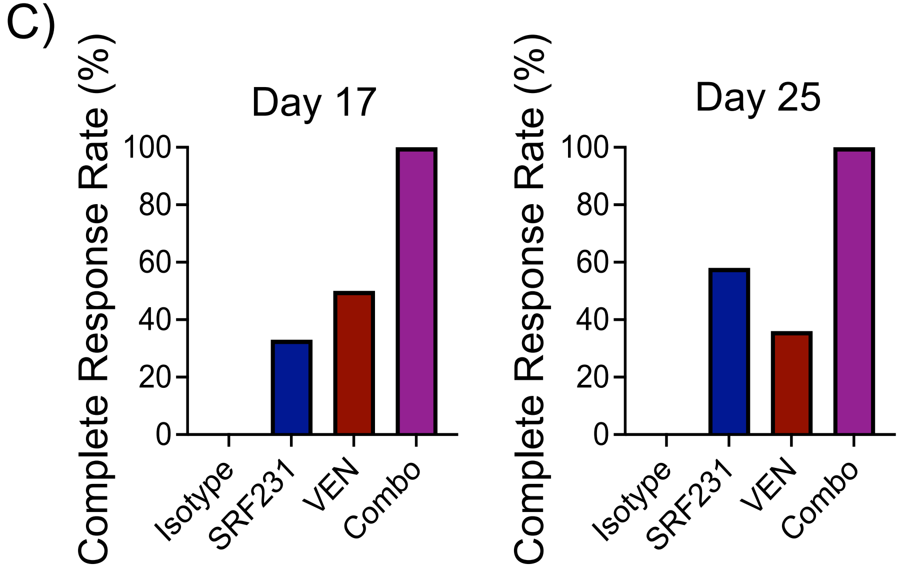

Day 17		
Group	CR's	Rate
Isotype	0 of 13	0
SRF231	4 of 12	33%
VEN	7 of 14	50%
Combo	14 of 14	100%

Day 25		
Group	CR's	Rate
Isotype	0 of 13	0
SRF231	7 of 12	58%
VEN	5 of 14	36%
Combo	14 of 14	100%

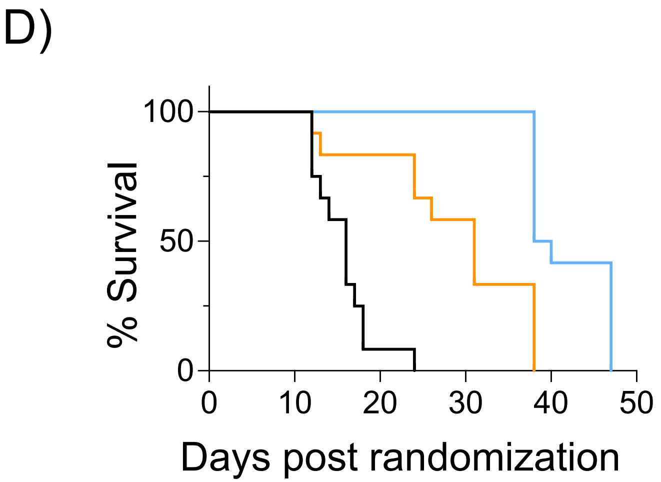

Supplementary Figure 8. In vivo findings of SRF231 and/or venetoclax-treated tumors of a BCL-2 dependent DLBCL xenograft model.

(A) Immunohistochemistry staining controls for phospho-MLKL (S358, T357, Skin) and phospho-RIP1K (S166, Thyroid Cancer).

(B) Line graph showing tumor volume (mm^3) on mice following treatment with isotype IgG4, SRF231 or venetoclax.

(C) Bar graphs and tables showing the complete response (CR) rates and actual mouse number with complete response, respectively, at day 17 and 25 for all treatment groups.

(D) Line graph showing survival of xenograft mice following treatment with isotype IgG4, SRF231 or venetoclax. Color-coded labels are similar to those of (B).

Supplemental Figure 9

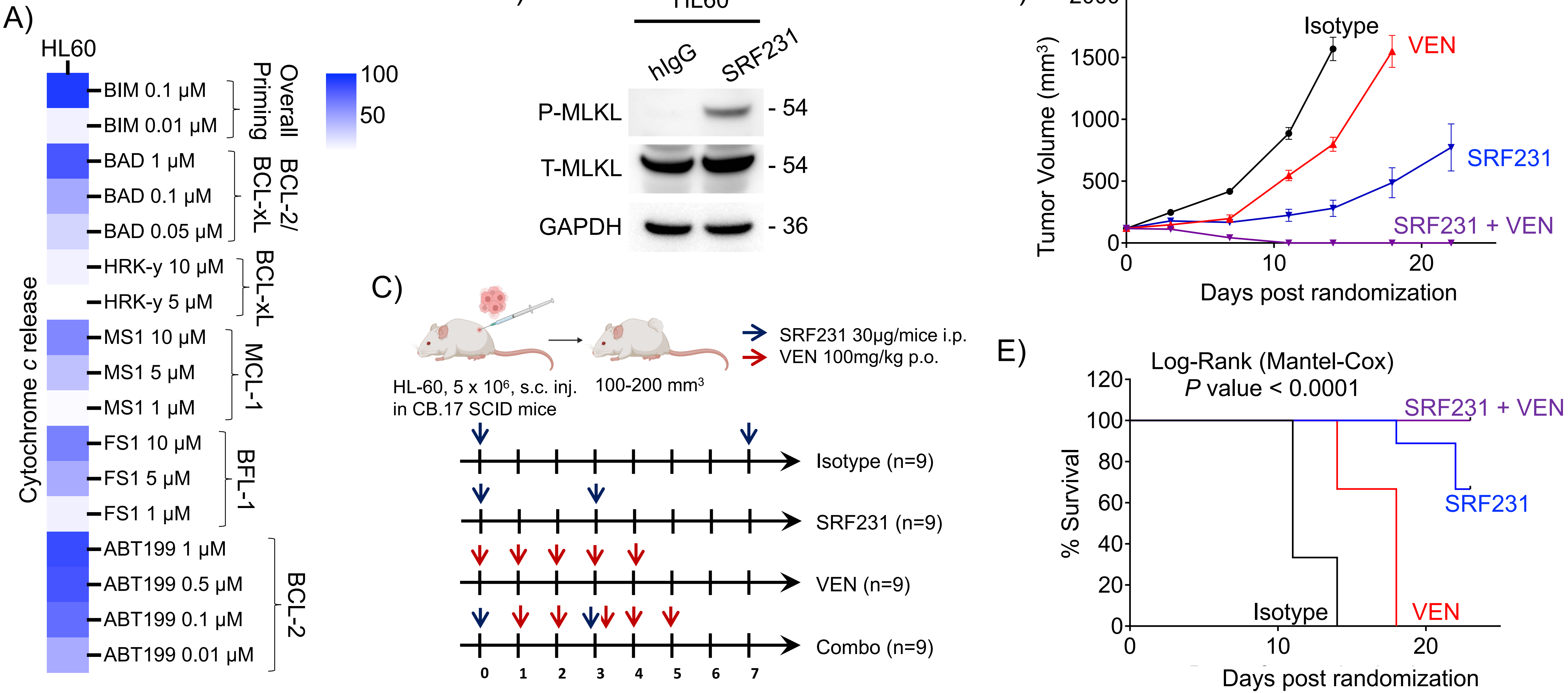

Supplementary figure 9. SRF231 is highly active in combination with venetoclax in a BCL-2 dependent AML xenograft model.

(A) BH3 profiling displays anti-apoptotic dependences of myeloid HL60. Right side labels indicating specific anti-apoptotic protein dependences.

(B) Western Blot showing p-MLKL following treatment with Protein G-bound hIgG or SRF231 (10 μg/ml, 24 hours) of HL60 cells.

(C) Illustration of the 4 treatment regimens. Treatments were performed for one cycle. Created in BioRender. Chamberlain, S. (2026) https://BioRender.com/f50pp7r.

(D) Line graph displays mean tumor volumes ± SEM. **** Combo vs. Isotype/VEN, * Combo vs SRF231 at Day 14, calculated by Sidak's multiple comparison test.

(E) Kaplan-Meier plots of overall survival of HL60 tumor-bearing animals treated with SRF231 +/- VEN. Statistics were calculated by Log-rank (Mantel-Cox) test.

Supplemental Figure 10

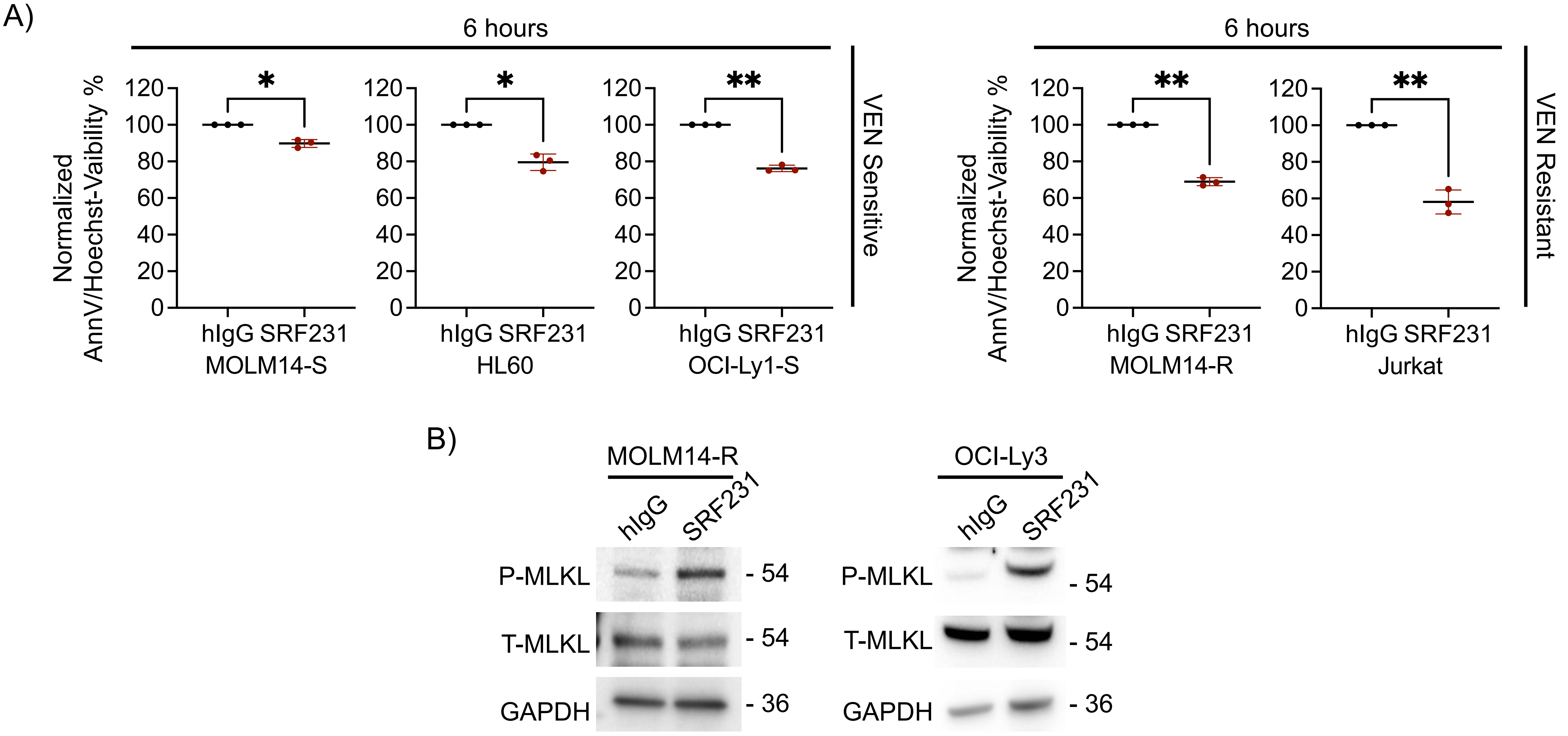

Supplementary Figure 10. SRF231 induces necroptotic-cell death at varying degrees in venetoclax sensitive and resistant cell lines.

(A) Cell death inductions of MOLM14-S (n=3), HL60 (n=3), OCI-Ly1-S (n=3), MOLM14-R (n=3), Jurkat (n=3) cells were measured following Protein G-bound SRF231 incubation with AnnV/Hoechst assay for 6 hours. Reported *P* values were calculated by paired Student's *t* test.

(B) Western Blot showing the increased p-MLKL (S358) after Protein G-bound SRF231 treatment of MOLM14-R and OCI-Ly3 cells (10 μg/ml, 6 hours).
